# Polymer theranostics with multiple stimuli-based activation of photodynamic therapy and tumor imaging

**DOI:** 10.7150/thno.86211

**Published:** 2023-09-04

**Authors:** Marina Rodrigues Tavares, Rayhanul Islam, Vladimír Šubr, Steffen Hackbarth, Shanghui Gao, Kai Yang, Volodymyr Lobaz, Jun Fang, Tomáš Etrych

**Affiliations:** 1 Institute of Macromolecular Chemistry, Czech Academy of Sciences, Heyrovského nám. 2, 16200 Prague, Czech Republic.; 2 Laboratory of Microbiology and Oncology, Faculty of Pharmaceutical Sciences, Sojo University, Kumamoto 860-0082, Japan.; 3 Institute of Physics, Photobiophysics, Humboldt University of Berlin, Newtonstr. 15, 12489 Berlin, Germany.

**Keywords:** fluorescence imaging, photodynamic therapy, pH-responsive theranostics, HPMA polymers, tumor-targeted nanomedicines

## Abstract

**Background:** Efficient theranostic strategies concurrently bring and use both the therapeutic and diagnostic features, serving as a cutting-edge tool to combat advanced cancers.

**Goals of the Investigation:** Here, we develop stimuli-sensitive theranostics consisting of tailored copolymers forming micellar conjugates carrying pyropheophorbide-a (PyF) attached by pH-sensitive hydrazone bonds, thus enabling the tumor microenvironment-sensitive activation of the photodynamic therapy (PDT) effect, fluorescence or phosphorescence.

**Results:** The nanomedicines show superior anti-tumor PDT efficacy and huge tumor-imaging potential, while reducing their accumulation, and potentially side effects, in the liver and spleen. The developed theranostics exhibit clear selective tumor accumulation at high levels in the mouse sarcoma S180 tumor model with almost no PyF found in the healthy tissues after 48 h. Once in the tumor, illumination at λ_exc_ = 420 nm reaches the therapeutic effect due to the ^1^O_2_ generation. Indeed, an almost complete inhibition of tumor growth is observed up to 18 days after the treatment.

**Conclusion:** The clear benefit of the specific PyF release and activation in the acidic tumor environment for the targeted delivery and tissue distribution dynamics was proved. Conjugates carrying pyropheophorbide-a (PyF) attached by pH-sensitive hydrazone bonds showed their excellent antitumor PDT effect and its applicability as advanced theranostics at very low dose of PyF.

## Introduction

Photodynamic therapy (PDT) has received considerable attention for the treatment of solid tumors 1, 2. PDT uses light as external stimuli to activate photosensitizer molecules (PSs) and oxygen to generate cytotoxic reactive oxygen species (ROS) such as peroxide, singlet oxygen (^1^O_2_), and hydroxyl species 3, 4. ROS directly kill the tumor cell by necrosis and apoptosis, and induce an inflammatory and immune response 5. Indeed, ^1^O_2_ is the primary toxic photochemical product, which, after the light irradiation of the PS with an appropriate wavelength, results in damage to DNA, RNA, proteins, and lipids, leading to cell death 5-9. Moreover, most of the PSs possess luminescence, which can be advantageously used for tumor imaging 5-8. The fluorescence imaging becomes in recent decade extensively studied modality for the preclinical development of various nanomedicines, novel method as such. Not only the diagnostic potential of the tumor accumulation but also the visualization of tumors during fluorescence-guided surgery could be achieved, 10, 11 in the best case, allowing the combination of fluorescence-guided resection and intraoperative PDT 12, 13.

There is considerable evidence for the use of PDT in the treatment of numerous solid tumors 5, 14, such as breast 15, prostate 16, ovarian 17, 18, bladder 19, 20, skin 21, and head and neck 22 cancers. Furthermore, few PSs have been approved for clinical trials, e.g., 5-aminolevulinic acid (5-ALA) or its methyl ester 23, 24, or even for clinical practice, such as porfimer sodium (Photofrin, Pfizer Japan Inc., Japan) for the treatment of early-stage lung 24, 25, esophageal, gastric, and early-stage cervical cancers 26, 27, talaporfin sodium (Laserphyrin, Meiji Seika Pharma Co., Ltd., Japan) for the treatment of early lung cancer 28, 29, and Temoporfin (Foscan, Biolitec Germany) for the treatment of squamous cell carcinoma of the head and neck 30. However, extant literature has reported several drawbacks concerning the use of common PSs, such as porphyrin derivatives, phtalocyanines, naphtalocyanines, and chlorins 31-34. Besides their low solubility in aqueous media due to their hydrophobic character, these low-molecular-weight compounds show poor selectivity and accumulation in tumors; therefore, they are distributed all over the body, resulting in side effects, such as cutaneous phototoxicity 35 for up to 6-8 weeks after treatment 36, and limiting both tumor-imaging intensity and therapeutic efficacy 6.

Tumor-targeted polymer delivery systems have gained attention as a strategy to overcome PS limitations and improve their pharmacokinetic properties, such as plasma half-life, and therapeutic efficacy owing to the enhanced permeability and retention (EPR) effect. The principle of the EPR effect relies on the leaky vasculature of tumor tissues and their vascular functions: tumor vasculature is characterized by large fenestrations between the endothelial cells formed during angiogenesis and by their irregular alignment. Moreover, they exhibit a dysfunctional lymphatic drainage, leading to the preferential extravasation/permeation into the tumor vasculature endothelium and the retention of macromolecules in the malignant tissue rather than the healthy one 37-39.

The encapsulation of several PSs into macromolecular systems, such as polymer nanoparticles 40-43 and polymer micelles 44, 45 and their target delivery has been reported. The combination of chemo-photodynamic synergistic cancer therapy based on nanoparticles (NPs) with encapsulated anticancer drug paclitaxel (PTX) and photosensitizer chlorin e6 has been developed to improve the therapeutic effect and decrease the systemic toxicity of current treatments. H_2_O_2_-responsive polymer prodrug NPs with GSH-scavenger were designed and prevented the depletion of ^1^O_2_, which induced the cooperatively strong oxidative stress and enhanced cancer cell apoptosis 46, 47.

Similarly, the covalent attachment of PSs to biocompatible and water-soluble polymer carriers forming polymer conjugates, which enhance the aqueous solubility of PSs, was described recently 48. The PDT effect is then localized mainly at the target tissue by the modulation of their biodistribution profile and higher accumulation in the tumor, resulting in efficient tumor imaging with a low background due to the lower accumulation into the normal tissues, and in a higher anti-tumor therapeutic effect 6. Naturally occurring porphyrins and chlorins are attractive photosensitizers, among them carboxyl-containing pyropheophorbide-a (PyF) is ideal for structural modification and conjugation to other biologically active molecules and hydrophilic polymer carriers, such as pHPMA. It was shown, that PyF methyl ester prepared by modification of 17C/carboxylic group of PyF showed higher photoinduced cytotoxicity than PyF 49.

Water-soluble and synthetic *N*-(2-hydroxypropyl)methacrylamide copolymers (pHPMA) have been extensively studied as carriers of bioactive molecules 50, 51. Also, some pHPMA-based drug conjugates have already been approved for clinical trials 52, 53. Recently, the pHPMA micellar conjugates of zinc protoporphyrin IX 7, 48 and pyropheophorbide-a (PyF) 6 bound by stable amide bonds were studied and were shown to have a remarkable antitumor effect and to be highly sensitive tumor imaging after selective accumulation in the tumor and ^1^O_2_ generation, making them promising theranostic candidates. A combination of the pHPMA conjugate of PyF with vascular mediators, as nitric oxide and carbon monoxide 54, 55, resulted in an increased anticancer effect in the different transplanted solid tumor models and pretreatment with the intralipid reduced liver uptake of a PyF conjugate and increased accumulation in the tumor 56. Few studies have focused on systems with releasable PSs responsive to internal triggers, such as pH-sensitive hydrazones 57, 58 or glutathione-sensitive disulfide bonds 59, 60.

Herein, we designed, synthesized, and characterized micelle-forming pHPMA-based conjugates with PyF 5-hydroxy-2-pentanone ester (dPyF) covalently attached by the pH-sensitive hydrolytically degradable hydrazone bond. The stimulus sensitivity of these potential theranostics was successfully proved. The highly improved tumor-targeted delivery of dPyF was observed by fluorescence imaging. The pH-responsiveness and controlled activation of dPyF fluorescence and singlet oxygen production enhanced the treatment outcome with very low doses of polymer theranostics.

## Experimental Section

### Materials

Chemicals such as 1-amino-propan-2-ol, 2,2′-azo*bis*isobutyronitrile (AIBN), methacryloyl chloride, 6-amino hexanoic acid, *tert*-butoxycarbonyl hydrazide, *N*-ethyl-*N*′-(3-dimethylaminopropyl)carbodiimide hydrochloride (EDC), carbon disulfide, ethanethiol, sodium hydride (60% dispersion in mineral oil), 5-hydroxy-2-pentanone, 4,6-trinitrobenzene-1-sulfonic acid (TNBSA), pentafluorophenol, 4-(dimethylamino)pyridine (DMAP), *tert*-butanol, *N,N*-dimethylacetamide (DMA), dichloromethane (DCM), dimethyl sulfoxide (DMSO), and 4-oxo-2,2,6,6-tetramethyl-1-piperidinyloxy, free radical (4-oxo-TEMP) were obtained from Merck (Czech Republic). Pyropheophorbide-a was purchased from Frontier Scientific^®^ (USA), 2,2′-azo*bis*(4-methoxy-2,4-dimethylvaleronitrile) (V-70) from Wako Chemicals (Germany), and *N*-(3-*tert*-butoxycarbonyl-aminopropyl)methacrylamide (APMA-Boc) from Polysciences, Inc., (USA). All of the other chemicals and solvents were of analytical grade. The solvents for the nuclear magnetic resonance (NMR) characterization of DMSO-*d*_6_ (99.80 atom% D) and CD_3_OD (99.80 atom% D) were obtained from VWR Chemicals (Belgium).

### Synthesis of pyropheophorbide-a derivative (dPyF)

**Figure 1:**
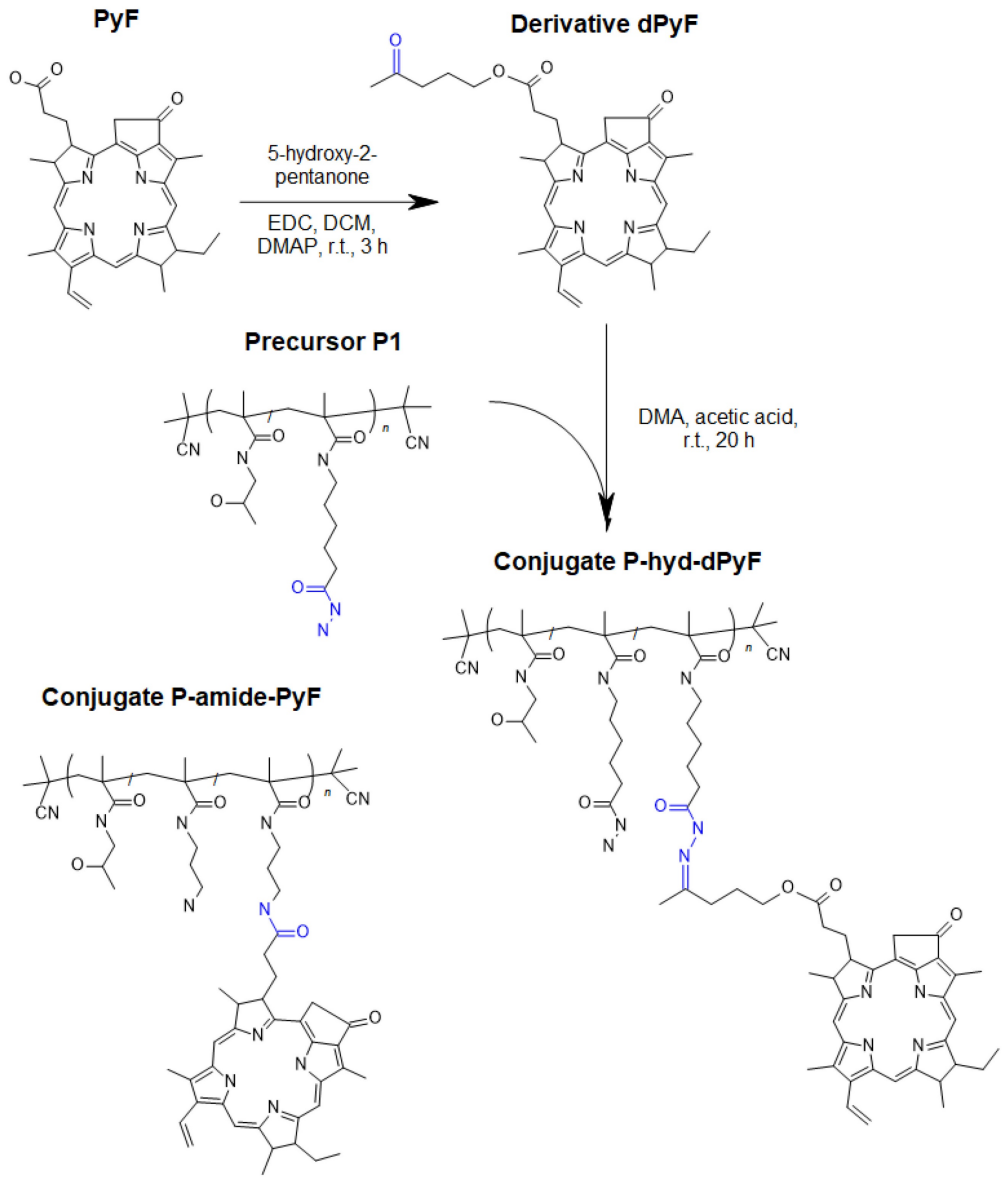
Scheme for the synthesis of dPyF and polymer conjugate **P-hyd-dPyF** containing dPyF bound by the pH-sensitive hydrazone bond. The schematic structure of the control polymer conjugate **P-amide-PyF** is depicted as well.

Carbodiimide chemistry was used to prepare a derivative of PyF (dPyF) via the esterification of the PyF carboxylic group with the hydroxy groups of 5‑hydroxy-2-pentanone (**Figure 1**). The purity of the dPyF was confirmed by HPLC and ^1^H NMR, and the molar absorption coefficient was determined by UV-Vis spectrometry. The synthesis of the pentafluorophenyl ester derivative of PyF to prepare polymer conjugates with PyF bound by stable amide bonds was presented in our previous study.^6^

The synthesis of dPyF was as follows: PyF (30 mg, 56.1 µmol) and *N*‑(3‑dimethylaminopropyl)-*N'*-ethylcarbodiimide hydrochloride (EDC) (16.1 mg, 84.2 µmol) were dissolved in DCM (4 mL). Then, 5-hydroxy-2-pentanone (8.5 µL, 84.2 µmol) and the catalyst 4-(dimethylamino)pyridine (DMAP) were added, and the reaction mixture was continuously stirred for 3 h. The reaction was monitored using TLC (CHCl_3_/MeOH, 20/1* v/v*), and the product was purified by a silica gel column (60 Å) using a gradient of CHCl_3_/MeOH, from 100/1 to 1/1* v/v*, elution) (34 mg, 98%). ^1^H NMR (CDCl_3_, 600 MHz): *δ* = 9.37, 9.28, and 8.57 ppm (3H, 3 × -C-C*H*=C- from PyF); *δ* = 7.91 ppm (1H, -C*H*=CH_2_ from PyF); *δ* = 6.24-6.12 ppm (2H, -CH=C*H*_2_ from PyF); *δ* = 5.26-5.08 ppm (2H, -C*H*_2_-C(O)- from PyF); *δ* = 4.48 ppm (1H, -C*H*-CH_3_ from PyF); *δ* = 3.97-3.89 ppm (2H, -C*H*_2_-O-C(O)- from aliphatic linker); *δ* = 3.13 ppm (3H, C*H*_3_-C(O)- from aliphatic linker); and *δ* = 0.26 and 1.81 ppm (2H, 2 × -N*H*- from PyF).

### Synthesis of monomers and chain transfer agent

Monomers *N*-(2-hydroxypropyl)methacrylamide (HPMA) and *N*-(*tert*-butoxycarbonyl)-*N'*-(6-methacrylamidohexanoyl)hydrazine (MA-AH-NHNH-Boc) were synthesized according to the literature 61, 62. The chain transfer agent (CTA) S-2-cyano-2-propyl S′-ethyl trithiocarbonate was synthesized as previously described 63. Their purity was assessed using HPLC, elemental analysis, and nuclear magnetic resonance (^1^H NMR) (Supporting Information).

### Synthesis of polymer precursors

The polymer precursor **P1** bearing hydrazide groups poly(HPMA-*co*-MA-AH-NHNH_2_) was prepared by the controlled radical reversible addition-fragmentation chain transfer (RAFT) copolymerization of HPMA and MA-AH-NHNH-Boc using S-2-cyano-2-propyl-S`-ethyl trithiocarbonate as a chain transfer agent (CTA) and the initiator 2,2ʼ-azo*bis*(4-methoxy-2,4-dimethylvaleronitrile) (V-70). The molar ratio of monomer/CTA/V‑70 was 360/2/1, and the molar ratio of monomers HPMA/MA-AH-NHNH-Boc was 90/10. Trithiocarbonate end groups were removed via reaction with an excess of 2,2′-azo*bis*isobutyronitrile (AIBN), and Boc groups were thermally removed in Q-H_2_O as previously described 64, 65. The detailed synthetic procedure for the synthesis of the polymer precursor **P1** is described in Supporting Information (**Figure S1**).

The polymer precursor **P2** containing amine groups poly(HPMA-*co*-MA-APMA) was prepared analogously by using HPMA and *N*-(3-*tert*-butoxycarbonyl-aminopropyl)methacrylamide (APMA-Boc), as previously described 6. The molar ratio of monomer/CTA/V‑70 was 500/2/1, and the molar ratio of monomers HPMA/APMA-Boc was 92/8. The physicochemical characterization of all of the polymer precursors is given in **Table 1**.

### Synthesis of polymer conjugates

The hydrazide groups of polymer precursor **P1** were reacted with the dPyF in DMA in the presence of acetic acid overnight in the dark, resulting in polymer conjugates with dPyF bound by hydrolytically cleavable hydrazone bonds (**P‑hyd-dPyF**) (**Figure 1**). The purity of the conjugate was assessed using ^1^H NMR, and TLC was performed in a mixture of CHCl_3_ and methanol (20/1, *v/v*). The synthesis of **P-hyd-dPyF** containing a 5-hydroxy-2-pentanone spacer was as follows: The polymer precursor **P1** (229 mg, 131.9 µmol hydrazide groups) and the dPyF (25 mg, 40.4 µmol) were dissolved in 3 mL of dry DMA. Then, 46 µL of acetic acid was added to the reaction, and the reaction mixture was continuously stirred at room temperature overnight in the dark. The conjugate was isolated and purified by precipitation into a mixture of ethyl acetate/CHCl_3_ (5/2, *v/v*, 200 mL) three times. The precipitate was filtered off, washed twice with a mixture of ethyl acetate/diethyl ether (1/1, *v/v*), and dried under vacuum (210 mg, 83%).

The polymer conjugate with PyF bound by stable amide bonds (**P-amide-PyF**) was prepared using the polymer precursor **P2** as previously reported 6 and used for comparison with the hydrolytically cleavable conjugate. The physicochemical characterization of all the polymer conjugates is given in **Table 2**.

### High-performance liquid chromatography

High-performance liquid chromatography (HPLC) was used to assess the purity of monomers, chain transfer agent, and dPyF, and to monitor the rate of dPyF release from the conjugate **P-hyd-dPyF**. A HPLC LC10 system equipped with a diode array detector SPD-M20A (Shimadzu, Japan) and a C18 reversed-phase Chromolith Performance RP‑18e column (150 mm, Merck, Germany) was used. Water/acetonitrile/0.1% TFA with a gradient of 5%-95% (*v/v*) acetonitrile at a flow rate of 4 mL min^-1^ was used as the eluent.

### Dynamic light scattering

Dynamic light scattering (DLS) was used to measure the hydrodynamic diameters (*D*_H_) of polymer precursors **P1** and **P2**, and polymer conjugates **P-hyd-dPyF** and **P-amide-PyF** (at λ = 632.8 nm, θ = 173°). In the case of conjugates, a fluorescence filter was used for the measurements (Zetasizer Ultra, Malvern Panalytical Ltd., United Kingdom). Data were evaluated using the DTS(Nano) software, and five independent measurements were performed using samples from 1 to 3 mg mL^-1^ in PBS at pH 7.4 (filter 0.22 µm PVDF).

### Size exclusion chromatography

The number-average molecular weight (*M*_n_), the weight-average molecular weight (*M*_w_), and the dispersity (*Đ*) of polymer precursors **P1** and **P2** were determined by size exclusion chromatography (SEC) using a HPLC LC40 system equipped with SPD-M40 photodiode array (Shimadzu, Japan), PSS Gram columns in series (PSS GRAM Lux analytical 30A and 2×PSS GRAM Lux analytical 1000 Å), Optilab‑rEX differential refractometer and DAWN HELEOS II multi-angle light scattering (MALS) detectors, (both Wyatt Technology Co., USA). The analysis was performed using DMF + LiBr (0.5 g L^-1^) as a mobile phase at a flow rate of 1 mL min^-1^. The data were analyzed using the ASTRA VI software, and the refractive index increment *dn/dc* (for PHPMA copolymers in DMF ~0.09 mL g^-1^) was applied for the calculation of *M*_n_, *M*_w_, and *Ð*.

### Field flow fractionation

Field flow fractionation (FFF) was applied to evaluate the stability of the micelles formed when the conjugate** P-hyd-dPyF** was prepared. Measurements were performed in duplicate by using the sample at 1.1 mg mL^-1^ in PBS (pH 7.4) at a flow rate of 1.0 mL min^-1^ by using an FFF (system Eclipse 3+) equipped with UV, DLS (DAWN Helios 8+), and Optilab-rEX differential refractometer index (RI) (Wyatt Technology Co., USA) detectors at λ = 658 nm. The ASTRA VI software was used, and the refractive index increment *dn/dc* (for pHPMA copolymers: ~0.167 mL g^-1^) was applied for the calculation of *M*_n_, *M*_w_, and *Ð* of the precursor.

### UV-Vis spectrophotometry

The molar content of hydrazide and amine groups statistically distributed along the polymer precursors **P1** and **P2**, respectively, were determined in the borate buffer by using the 2,4,6-trinitrobenzene-1-sulfonic acid (TNBSA) assay method described before 66. The molar absorption coefficients used were *ε*(NHNH_2_) = 17,200 L mol^-1^ cm^-1^ (λ_max_ = 500 nm) for hydrazides and *ε*(NH_2_) = 11,550 L mol^-1^ cm^-1^ (λ_max_ = 420 nm) for amines. The amount (wt.%) of dPyF and PyF in the conjugates was assessed in methanol by using the following molar absorption coefficients: in the case of **P‑hyd‑dPyF**, *ε* = 69,100 L mol^-1^ cm^-1^ (λ_max_ = 416 nm) and 32,200 L mol^-1^ cm^-1^ (λ_max_ = 668 nm) determined for the dPyF; in the case of **P‑amide‑PyF**, *ε* = 105,700 L mol^-1^ cm^-1^ (λ_max_ = 416 nm) and 45,000 L mol^-1^ cm^-1^ (λ_max_ = 668 nm) determined for the PyF compound. The samples' absorbance spectra were measured at 0.1 mg mL^-1^ PyF equivalent in methanol and recorded from 200 to 800 nm by using Specord 205 ST (Analytic Jena AG, Germany). The absorption spectra of PyF, dPyF and polymer conjugates are shown in Figure S2.3.

### Nuclear magnetic resonance

^1^H NMR spectra were measured using a Bruker AVANCE III 600 spectrometer operating at 600.2 MHz by using DMSO-*d*_6_ for monomers and polymer conjugates, or CDCl_3_ for dPyF. To compare the molar content of dPyF in the conjugates with the results obtained by UV-Vis, the molar content of PyF in the polymer conjugates was calculated using the integral intensity of signals at *δ* = 6.23 ppm (1H, -CH=C*H*_2_) and *δ* = 6.42 ppm (1H, -CH=C*H*_2_) from the PyF molecule, and *δ* = 3.67 ppm (1H, C*H*-OH) or 4.71 ppm (1H, CH-O*H*) of the HPMA monomer unit. The ^1^H NMR spectra of monomers and polymers are listed in Figures S2.1.1 - S2.1.8.

### Transmission electron microscopy

The morphology and dimensions of P-hyd-dPyF were examined using transmission electron microscopy (TEM). A 10 mg/ml solution of P-hyd-dPyF was prepared in deionized water. Subsequently, 50 μl of the solution was combined with 50 μl of 0.01% phosphotungstic acid for TEM analysis, using a JEM-1400 plus microscope manufactured by JEOL in Tokyo, Japan. The TEM image of P-hyd-dPyF is shown in Figure S2.4.

### dPyF release from polymer conjugate P-hyd-dPyF

The amount of dPyF released from the polymer conjugate **P-hyd-dPyF** was monitored by incubating the sample in a 0.1M phosphate buffer solution with 0.05M NaCl at pH values of 5.0 and 7.4 at 37°C, followed by extraction in chloroform as previously reported 67. The released amount of dPyF was diluted in a mixture of methanol/DCM (3/2,* v/v*) and determined using an HPLC analysis. The relative area of peaks (absorbance at λ = 416 nm) was used for the calculation, and all of the drug-release data were expressed as the amount of free dPyF relative to the total dPyF content in the conjugate. All of the experiments were carried out in triplicate.

### Fluorescence spectroscopy

The fluorescence spectra of pure PyF, **P-hyd-dPyF**, and **P-amide-PyF** were recorded using a spectrofluorometer model FP-6600, Jasco Corp., Tokyo, Japan. **P-hyd-dPyF** was dissolved in PBS in the absence/presence of the indicated concentrations of Tween-20, sodium dodecyl sulfate (SDS), lecithin, and urea. For comparison between polymer conjugates **P-hyd-dPyF** and **P-amide-PyF** with pure PyF, the samples were dissolved in PBS (pH 7.4) or in ethanol, and **P‑hyd-dPyF** was incubated in PBS (pH 5.0) with SDS 0.1% (*w/v*) at 37°C for 4 h. The sample solutions were prepared at 0.005 mg mL^-1^ dPyF equivalent, excited at λ_max_ = 420 nm (corresponding to PyF), and the emission from 600 to 800 nm was recorded.

### Isothermal titration calorimetry (ITC)

The solutions of **P-hyd-dPyF** and **P-amide-PyF** polymers in PBS were titrated to pure PBS at 37°C in 20 consecutive injections using a MicroCal ITC200 isothermal titration calorimeter (Malvern Panalytical Ltd, UK). Additionally, blank titrations of **P1** and **P2** polymer precursors were performed in a similar manner. The heat for each injection (µJ) was plotted against the concentration of the polymer in the cell after the corresponding injection (Figure S2.5). The concentrations of polymers before dilution were adjusted to 1 and 10 mg mL^-1^, respectively. The concentration ranges during the dilution were 0.011-0.16 and 0.11-1.7 mg mL^-1^ for 1 and 10 mg mL ^-1^ initial solutions, respectively. To determine the contribution of the porphyrin aggregate (stack, micelle core) dissociation to the overall heat of dilution, the heat from blank titration was subtracted from the heat of dilution of the polymer-porphyrin conjugate. Finally, the pH of all initial solutions was measured to account for the potential contribution of (de)protonation originating from the pH mismatch to the overall heat of dilution.

### Electron spin resonance spectroscopy

The ^1^O_2_ generation was measured using electron spin resonance (ESR) spectroscopy. Solutions of **P-hyd-dPyF** were prepared in the concentration of 40 μg mL^-1^ PyF equivalent in the following solvents: (a) PBS at pH 6.5, (b) PBS at pH 7.4, (c) PBS at pH 5.0, (d) solution of Tween-20 at 0.5% (*v/v*) in PBS pH 7.4 and (e) after incubation in PBS (pH 5.0) with Tween-20 at 0.5% (v*/v*) at 37°C for 4 h. For all of them, 5% (*v/v*) of ethanol was added for dissolution. Then, 100 μL of 200 mM 4-oxo-TEMP (spin trapping agent) was added to 900 μL of the sample (final concentration of 20 mM 4-oxo-TEMP), which was placed into flat quartz cells (Labotec, Tokyo, Japan). The illumination was carried out at different times (from 0 to 600 s) by using a xenon light source (MAX-303; Asahi Spectra Co. Ltd., Tokyo, Japan) from 400-700 nm. The X-band ESR spectra were recorded using a JEOL JES FA-100 spectrometer (Tokyo, Japan) at 25°C. The ESR spectrometer was usually set at a microwave power of 1.0 mW, amplitude of 100 kHz, and field modulation width of 0.1 mT.

### In vitro cytotoxicity assay

Mouse colon cancer C26 cells were maintained in RPMI-1640 and supplemented with 10% fetal bovine serum (FBS, Nichirei Biosciences Inc., Tokyo, Japan) under 5% CO_2_/air at 37°C. Cells were seeded in 96-well plates with 3000 cells per well, and after overnight preincubation, the polymer conjugates **P-hyd-dPyF**,** P-amide-PyF**, or** free PyF** were added at different concentrations. After 24 h treatment, media were removed, and the cells were washed three times with PBS followed by replacement with fresh media. Illumination was then carried out with fluorescent blue light with peak emission at 420 nm (TL-D; Philips, Eindhoven, Netherland) of 1.0 J cm^-2^. After further 24 h of culture, the MTT assay 68 was carried out to quantify viable cells.

### Measurement of intracellular ROS after P-hyd-dPyF treatment

The C26 cancer cells were seeded in 12-well plates at a density of 2 × 10^5^ cells/well and incubated overnight. Subsequently, different concentrations of P-hyd-dPyF were applied to the cells, and they were treated for 24 hours. To assess intracellular ROS, the cells were treated with the intracellular ROS probe 7-dichlorodihydrofluorescein diacetate (DCDFH-DA, Sigma) at a concentration of 10 μM and further cultured for 30 minutes. During this period, the esterified form of DCDFH-DA permeated the cell membranes and underwent deacetylation to form DCDHF, which could react with ROS to produce a fluorescent compound known as dichlorofluorescein. Following the incubation with the ROS probe, the cells were exposed to light irradiation as described above. Subsequently, the amount of intracellular ROS was quantitated by measuring the fluorescence intensity using flow cytometry (BD AccuriTM C6 Plus; Becton Dickinson, San Jose, CA, USA).

### Intracellular uptake of polymer conjugates in cultured cells and spheroids

The intracellular uptakes of polymer conjugates as well as free PyF were examined in the above-described C26 cells. The cells were seeded in 96-well plates (200,000 cells/well). After overnight preincubation, the polymer conjugates **P-hyd-dPyF** or **free PyF** were added (0.5 μmol, PyF equivalent). At the indicated time points after treatment, the media were removed, and the cells were washed three times with PBS followed by replacement with fresh media. Then, the cells were subjected to *in vivo* fluorescence imaging using IVIS XR (Caliper Life Science, Hopkinton, MA) with an excitation of 675 nm and an emission of 695-770 nm. In a separate experiment, cells were seeded in 12-well pates (50,000 cells/well), and after overnight preincubation, **P-hyd-dPyF** or **P-amide-PyF** were added at the concentration of 0.5 μmol (PyF equivalent). Two hours after treatment, the media were removed, and the cells were washed three times with PBS. Then, 1 mL of ethanol was added, and the cells were harvested and collected; the internalized polymer conjugates or free PyF were extracted using ethanol under sonication (30 W, 30 s, UP50H homogenizer, Hielscher Ultrasonics GmbH, Teltow, Germany) on ice and quantified using fluorescence spectroscopy (excitation at 420 nm, emission at 650 nm).

Further, the internalization and penetration of polymer conjugates were investigated using C26 cell spheroids. C26 cells (2 × 10^5^) were seeded in 14 cm^2^ ultra-low attachment cell dishes (Corning Inc., Corning, NY, USA). After six days of culture, the formed cell spheroids were transferred into 9.6 cm^2^ glass-bottomed culture dishes. Polymer conjugates **P-hyd-dPyF**, **P-amide-PyF**, or** free PyF** were then added at a final concentration of 0.5 μmol (PyF equivalent), and at the scheduled time (4 h, 8 h) after treatment, the polymer conjugates or free PyF in the spheroids were visualized by confocal laser fluorescence microscopy (Nikon TE2000U, Nikon Solutions Co., Ltd., Tokyo, Japan) at the excitation wavelength of 405 nm and the emission wavelength of 570-640 nm.

### In vivo tissue distribution and in vivo fluorescence imaging

Six-week-old male ddY mice (30-35 g) were purchased from SLC, Shizuoka, Japan. The animals were housed under controlled conditions of 20-22°C and 45%-50% humidity with a 12 h light/dark cycle, with free access to water and food. All of the animal experiments were approved by the Animal Ethics Committees of Sojo University (no. 2020-P-009, approved on April 1, 2020) and carried out according to the Guidelines of the Laboratory Protocol of Animal Handling, Sojo University.

The mice were fed adaptively for one week before the experiments. Sarcoma S180 cells were implanted subcutaneously in the dorsal skin of ddY mice (2 × 10^6^ cells/0.1 mL). At 10-12 days after tumor inoculation, when the tumor reached approximately 10 mm, the polymer conjugate with the concentration of 5 mg kg^-1^ PyF equivalent in the physiological saline solution was injected into the tail vein. The mice were sacrificed at the scheduled time, e.g., 2 h, 4 h, 8 h, 24 h, and 48 h after intravenous administration, and then, the tumor and normal tissues (liver, spleen, kidneys, lung, heart, muscle, and colon) were dissected and weighted. Then, DMSO was added at the concentration of 1 mL per 100 mg of tissue. The tissues were homogenized and centrifuged at 12,000 RPM at 25°C for 15 min. Moreover, an aliquot of 1 mL of blood was centrifuged at 4,000 RPM for 15 min, and then, 50 µL of the plasma was taken and diluted in 450 µL of DMSO. The quantification of the extracted PyF was performed by fluorescence spectroscopy by the dilution of supernatants in DMSO (excitation of 420 nm and emission of 650 nm). The data were then analyzed using the fluorescence intensity areas. In some experiments, the human ovarian cancer xenograft model was used, wherein human ovarian cancer A2780 cells (1 × 10^7^) were implanted in the dorsal skin of the BALB/c nude mice (male, six weeks old, SLC). After 6-8 weeks when the tumors grew to 10-15 mm in diameter, **P-hyd-dPyF** was injected intravenously, and the *in vivo* tissue distribution assay and *in vivo* imaging were carried out using the same protocol as that described above. The amount of drug in each tissue was quantified using a standard curve of **P-hyd-dPyF**, which was added into tissue homogenates of the untreated control mice followed by the same extraction and measurement process as that described above.

In addition, in these experiments, the mice were subjected to *in vivo* imaging by using IVIS XR (Caliper Life Science, Hopkinton, MA, USA) before they were sacrificed, and the dissected tissues were subjected to fluorescence imaging by using the IVIS system and the above-described protocol (excitation of 675 nm and emission of 695-770 nm).

### In vivo PDT antitumor activity

The S180 solid tumor model, as described above, was used in this study. At 7-10 days after tumor inoculation when the tumor diameters reached approximately 8-10 mm, each polymer conjugate (**P-hyd-dPyF** or **P-amide-PyF**) was dissolved in physiological saline and administered intravenously at the indicated concentrations. The control mice were injected with physiological saline. At 24 and 48 h after the administration of the polymer conjugate, the tumor was illuminated by a xenon light (MAX-303; Asahi Spectra) at 400-700 nm for 5 min (27 J cm^-2^). The tumor volume (mm^3^) was calculated as (W^2^ × L)/2 by measuring the width (W) and length (L) of the tumor, and the body weight of the mice was measured during the study period. In some experiments, with a fractionated dose of **P-hyd-dPyF** (2.5 mg kg^-1^), two cycles of PDT were performed.

The same experiment was also conducted using C26 tumor-bearing mice. The tumor model was established by injecting C26 cells (2 × 10^6^ cells/0.1 mL) into the dorsal skin of Balb/c mice (male, 6-week old, SLC). In this study, the mice were sacrificed either 25 days after treatment or when the tumor reached a size of 2000 mm^3^. Subsequently, the liver and kidney were collected, and cryo-sections of 10 μm were prepared, followed by H&E staining, to evaluate the potential side effects of this treatment.

### Singlet oxygen and PyF phosphorescence detection in vivo

To follow the tumor accumulation of the systemically injected conjugate over time using NIR detection, the animal models described in Section 2.17 were used. We investigated the **P-hyd-dPyF** conjugate at a dose of PyF 5 mg kg^-1^ body weight in a physiological saline solution. After 6, 24, 48, and 72 h, the time-resolved NIR-luminescence was measured for 1 min each. The selected spectral region around 1270 nm contained the characteristic phosphorescence of singlet oxygen and the contributions of the long wavelength tail of the PyF phosphorescence as the main components. The setup consisted of a fiber-coupled Laserdiode Red65X (Necsel), driven in the pulsed mode (~350 ns/12 kHz) by a custom-built driver, based on the iC-HG30 (iC-Haus). The NIR detection system TCMPC1270 (SHB Analytics GmbH, Germany) was used with a fiber adapter and a custom-designed multi-furcated quartz fiber (Ceram Optec). The observed time window covered 80 µs, starting with excitation, at a channel width of 20 ns.

### Statistical analysis

The results were expressed as mean ± SD. Student's *t*-tests were performed to assess the differences between the two groups. Analysis of variance (ANOVA) followed by the Bonferroni *t*-test was used for comparison between groups. The difference was considered statistically significant when *P* < 0.05.

## Results and Discussion

**Figure 2:**
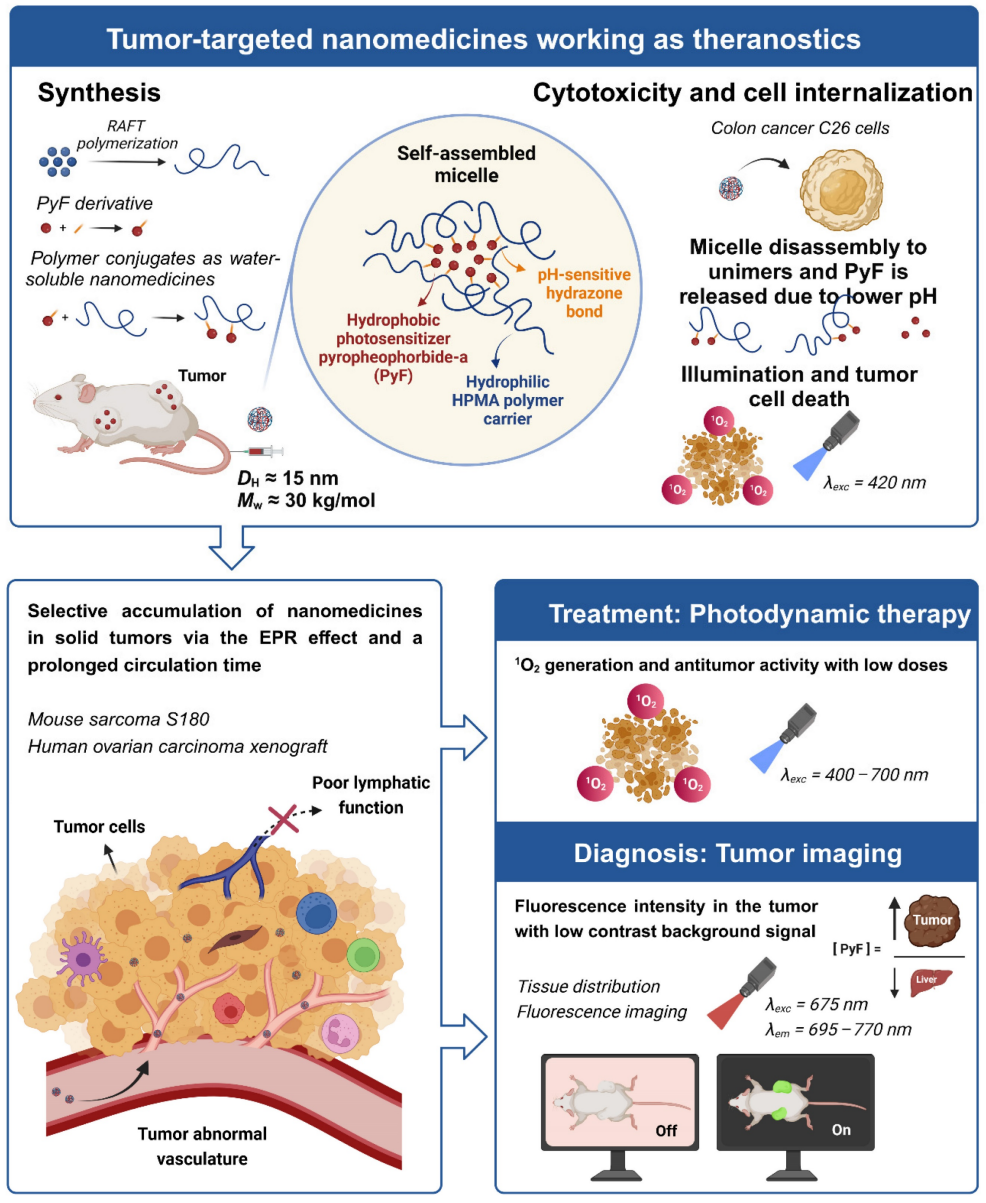
Overview showing the synthesis of tumor-targeted HPMA-based nanomedicines working as theranostics. The illustration depicts the selective accumulation of these micellar systems carrying PyF in solid tumors via the EPR effect. These conjugates fulfill the basic criteria for theranostics, combining therapy (^1^O_2_ generation and antitumor activity) with diagnosis (tumor imaging with high fluorescence in the tumor) with the advantage of a high tumor/liver accumulation ratio of PyF.

Nanomedicines exhibiting a theranostic nature are believed to be the next generation of advanced therapeutics. They can provide a real insight into the treatment's procedure and thus increase the therapy efficacy in real time by the modification of the dosing. Recently, we reported HPMA-based theranostics-bearing PyF bound by stable amide bonds and proved both the imaging potency and the PDT effect of these systems, which also showed prolonged circulation times and tumor-targeted accumulation based on the EPR effect 6. However, we were concerned about their high accumulation in the liver and the spleen, which are rich in the reticuloendothelial system (RES), which traps nanoparticles, and are the major organs to metabolize porphyrin derivatives. Therefore, we decided to use a pH-sensitive stimulus as a smart approach, which has been repeatedly proven to increase the therapeutic efficacy in tumor treatments.^69^ Herein, we designed, synthesized, and evaluated a stimuli-responsive hydrophilic copolymer conjugate **P-hyd-dPyF** as a tumor-targeted and pH-sensitive nanomedicine (**Figure 1**). In this study, we aimed for a specific drug release, following tumor cell uptake and reliable photosensitization in the tumor, while reducing the PS concentration in the liver and the spleen. The temporary dormant state of the PyF when bound to the polymer backbone forming self-assembled micelles is beneficial from the off-target activity point-of-view, as such a hydrophobic core-localized PyF was non-active before the disruption of the micellar systems and release from the polymer carrier (see schematic representation in **Figure 2**).

### Synthesis and physico-chemical characterization of polymer precursors and conjugates

The controlled RAFT polymerization technique was used to synthesize almost monodisperse polymer precursors with molecular weights below the limit of glomerular filtration 70. The linear polymer precursor **P1**, poly(HPMA-*co*-MA-AH-NHNH_2_), with hydrazide groups statistically distributed along the polymer chain showed molecular weights around 26⋅10^3^ g mol^-1^ and a dispersity below 1.05. Similarly, the linear polymer precursor **P2**, poly(HPMA-*co*-MA-APMA), with amine groups showed molecular weights around 30⋅10^3^ g mol^-1^ and a narrow dispersity (**Table 1**). In both of the cases, the number of functional groups was adjusted to be sufficient for the further attachment of the active PS moiety. **P1** hydrazide groups allowed the attachment of the dPyF up to 7 wt.%, forming the polymer conjugate **P-hyd-dPyF** with dPyF bound via the hydrolytically cleavable and pH-sensitive hydrazone bonds (**Figure 1**), and the control polymer conjugate **P-amide-PyF** was prepared from the precursor **P2** by the attachment of the PyF via an amide bond as described recently 6.

As expected, **P1** and **P2** showed the typical *D*_H_ around 9-10 nm for the linear HPMA-based polymer random coils in aqueous solutions, ensuring their safe elimination from the organism via renal filtration after the drug release 70. The GPC performed under the non-aqueous conditions proved that the attachment of PyF changed neither the molecular weight nor the dispersity (Supporting Information, **Figure S2.2**). Indeed, the attachment of PyF or its derivative dPyf to the polymer carrier significantly increased the hydrodynamic sizes for the polymer-PyF/dPyF conjugates in an aqueous solution, *D*_H_ ≈ 15 nm, indicating the macromolecules' self-assembly into micelles due to the amphiphilic character caused by the presence of the hydrophobic PS in their structures (**Table 2**). The TEM image also proved the micelle formation of P-hyd-dPyF showing a diameter of about 15 nm (Figure S2.4). Importantly, the sizes for both of the polymers were approximately the same, thus showing the same character of self-assembly properties. Each micelle consisted of a sufficiently stable hydrophobic core, which in our case meant that a sufficient number of dPyF or PyF molecules could interact in the micellar core. The micelles were stable for 5 days incubation in culture medium showing the applicability as theranostics.

As the hydrodynamic sizes of both polymer micelles were above the renal filtration threshold, their reduced urinary excretion and prolonged circulation in the organism could be expected, which is an advantage from the pharmacokinetics point of view. Moreover, the characteristics of these amphiphilic systems were additionally evaluated using FFF. In the case of precursor **P1**, only the single polymers, unimers, were found, and the molecular weight measured using FFF was *M*_w_ = 26⋅10^3^ g mol^-1^ (*Ð* = 1.1), which agreed with the SEC results. Indeed, the conjugate **P-hyd-dPyF** showed a very small fraction of unimers, less than 5%, indicating a predominant formation of stable micelles. The small amount of unimers can be ascribed to the presence of polymer chains without a dPyF molecule (Supporting Information, **Figure S2.2**). For the conjugates **P-hyd-dPyF** and **P-amide-PyF** no significant heat signature, which can be attributed to the dissociation of porphyrin stacks upon dilution was found until the concentration of 0.011 mg.mL^-1^ (Fig. S2.5). Although a non-zero exothermic heat of dilution was recorded, it originates from the dilution of the hydrophilic polymer carrier and almost vanishes after the subtraction of a blank. No measurable dissociation of porphyrin stacks is detected up to the concentration of 0.011 mg.mL^-1^ (detection limit of the method). We can conclude that both the polymer conjugates form a stable micelle, which CMC is below 0.011 mg. mL^-1^.

**Figure 3:**
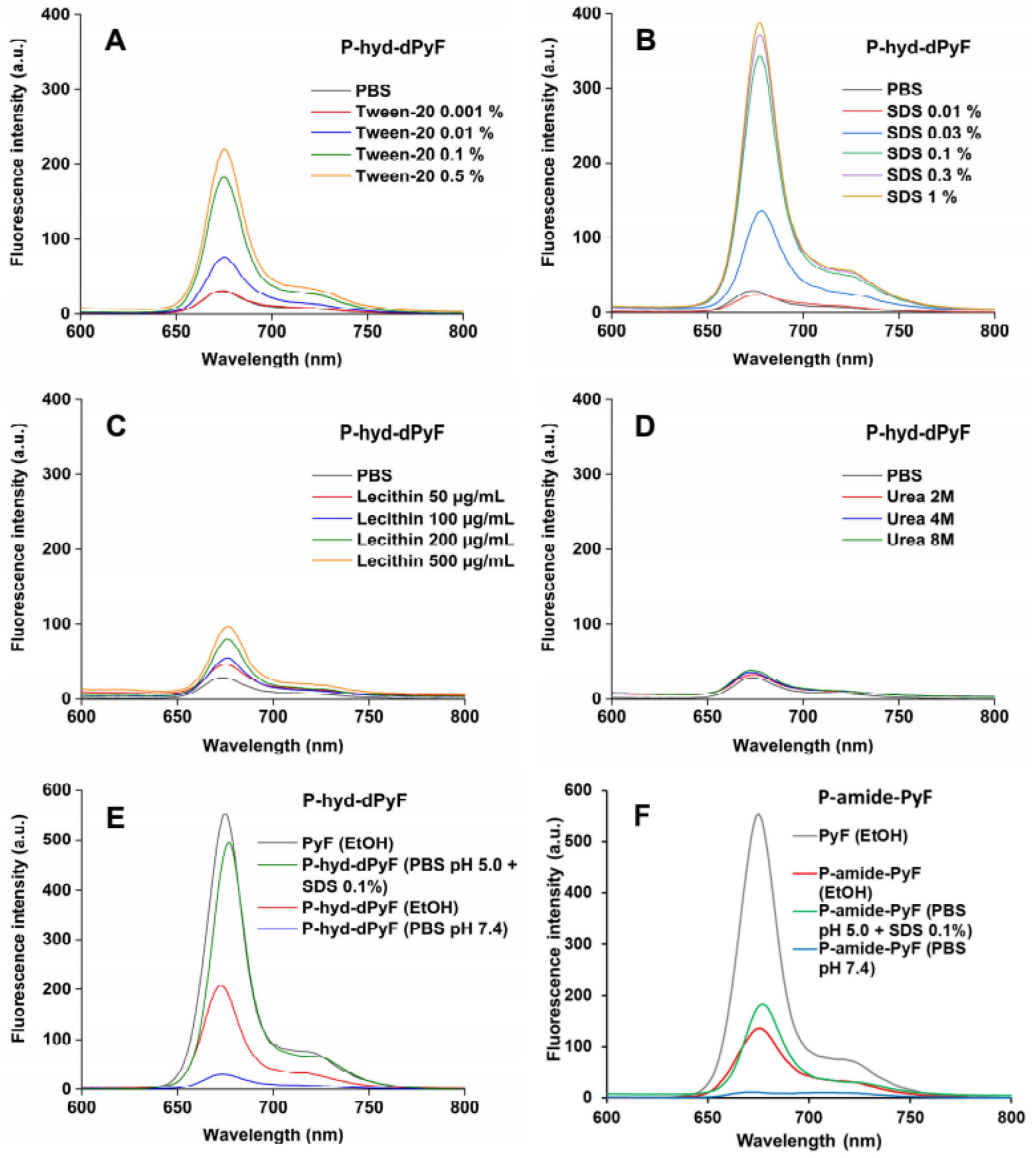
Fluorescence spectra of **P‑hyd-dPyF** in PBS in the absence/presence of Tween-20 **(A)**, sodium dodecyl sulfate (SDS)** (B)**, lecithin **(C)**, and urea **(D)**. Fluorescence spectra of pure PyF in EtOH compared with **P‑hyd-dPyF (E)** or** P‑amide-PyF (F)** in EtOH and PBS. All of the sample solutions were prepared at 0.005 mg mL^-1^ PyF or dPyF equivalent and excited at λ_max_ = 420 nm (corresponding to PyF).

We also investigated the fluorescence from the conjugate **P-hyd-dPyF** in the absence/presence of different compounds, such as Tween-20, SDS, lecithin, and urea (**Figures 3A-D**). In aqueous solution (i.e., PBS), we observed almost no fluorescence, indicating fluorescence quenching and suggesting the micelle formation of P-hyd-dPyF. However, in the presence of surfactants such as Tween-20 and SDS, which have the capability to disrupt micelle self-assembly, we observed strong fluorescence. Additionally, incubating the samples with urea resulted in no fluorescence. These findings indicate that hydrophobic interactions, rather than hydrogen bonds, play a crucial role in the micelle formation. Importantly, when lecithin, a lipid component found on cell membranes, was added, a significant increase in fluorescence was observed. This suggests that the micelle formation of P-hyd-dPyF is disrupted during intracellular uptake. These results provide valuable insights into the behavior of P-hyd-dPyF in different environments and in vivo scenarios. The disruption of micelle formation in the presence of specific surfactants and lecithin highlights the importance of understanding the interactions and behavior of P-hyd-dPyF in various biological settings.

**Figure 4:**
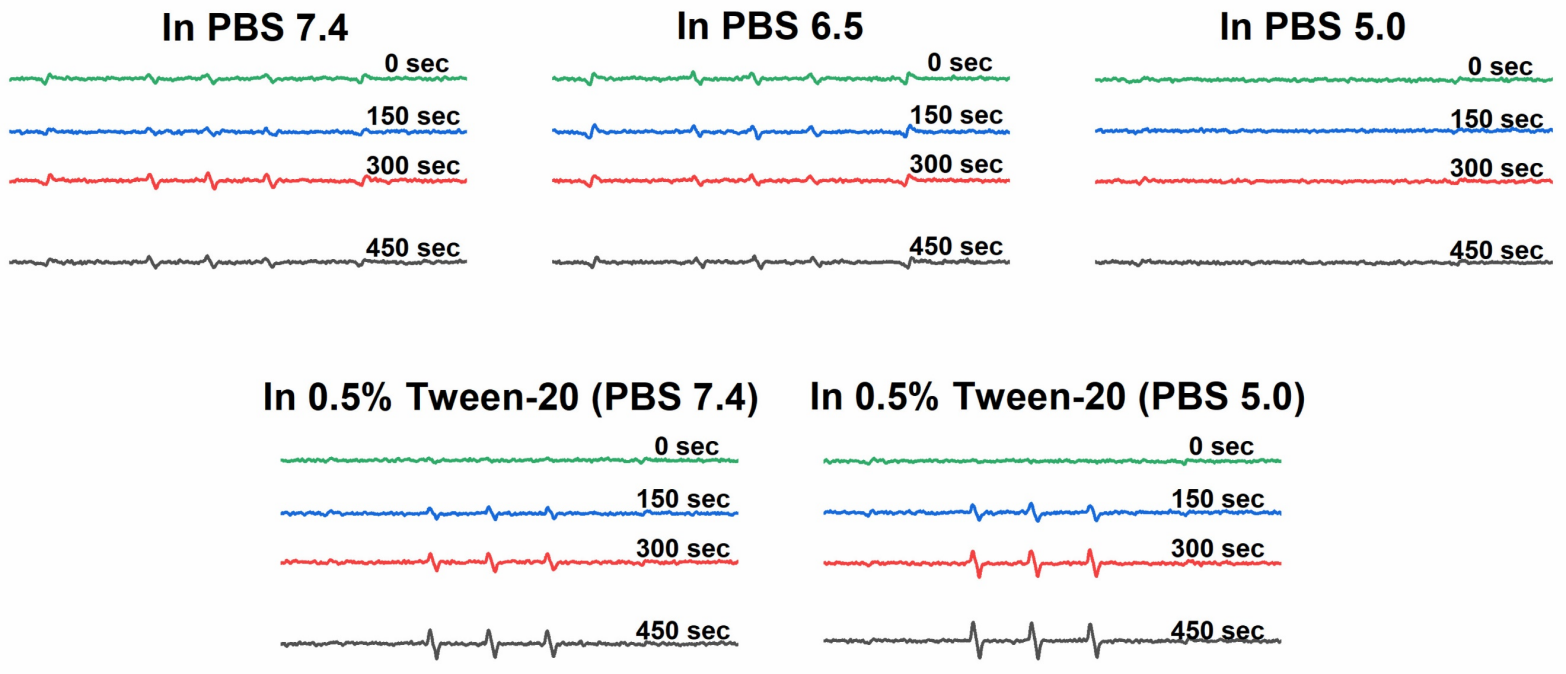
ESR spectra of ^1^O_2_ generation for **P-hyd-dPyF** in PBS at pH 6.5, PBS at pH 7.4, and Tween‑20 at 0.5% (*v/v*) in PBS (pH 7.4). Polymer conjugate solutions were prepared in the concentration of 40 μg mL^-1^ PyF equivalent, and 4-oxo-TEMP (20 mM) was used as the spin trapping agent. Illumination was carried out at the indicated time points by using a xenon light source from 400-700 nm.

The fluorescence of **P‑hyd-dPyF** increased by approximately eight times when Tween-20 was used at 0.5% (*v/v*), which was similar to the results published previously 6 for **P-amide-PyF** and increased by fourteen times when SDS was used at 0.3% (*w/v*). Taken together, these findings can be correlated with the lower generation of ^1^O_2_ in PBS even after illumination and the higher generation of ^1^O_2_ after the micelle disruption by Tween-20 (**Figure 4**), ensuring the safety of the micellar systems in circulation. Moreover, the attachment of PyF or dPyF to the polymer carriers decreased the fluorescence intensity in EtOH and almost depleted the intensity in PBS due to the excitonic interactions between PyF molecules distributed along the polymer backbone, confirming the tendency of PyF or dPyF to stack together and to form the hydrophobic core of micelles or aggregates in aqueous solutions (**Figures 3E-F**). We observed a similar behavior for the UV-Vis absorption spectrum, which indicates the depletion of absorption after the attachment of PyF to the polymer carrier 71 (**Figure S2.3**). More importantly, when **P-hyd-dPyF** was dissolved in PBS at pH 5.0 with SDS as the surfactant, the fluorescence increased to almost the same as that found for the PyF compound, which can be ascribed to the PyF release from the conjugate (**Figure 3E**), whereas P-amide-PyF showed much lower fluorescence at the same condition (**Figure 3F**). Such behavior may be highly important in the tumor microenvironment conditions* in vivo*. We hypothesized that, particularly for the **P-hyd-dPyF** conjugate investigated here where the polymer backbone carries several dPyF moieties, the release of dPyF in the tumor area could increase the fluorescence and possibly the singlet oxygen production.

### Generation of ^1^O_2_ from polymer conjugate P-hyd-dPyF

We found that the ^1^O_2_ generation from **P-hyd-dPyF** was suppressed in PBS at pH values of 6.5 and 7.4 even after 450 s of illumination, confirming the conjugate self-assembly and micelle formation in aqueous solutions. The ^1^O_2_ generation was depleted in the same manner as the fluorescence. We considered this an advantage, as the aggregation and thus the low activity of dPyF is highly welcome during circulation and transport to the tumor tissue. However, once in the tumor, the generation of ^1^O_2_ is necessary for effective PDT therapy; therefore, we diluted **P-hyd-dPyF** in a solution of Tween-20 in PBS 7.4 for comparison (**Figure 4**). Clearly, the addition of Tween-20, amphiphilic surfactant, increased the ^1^O_2_ generation dose-dependently, which was consistent with the fluorescence increase observed before. Here, the Tween-20 could separate the aggregated dPyF molecules into Tween-20-dPyF nanovesicles, leading to the disintegration of the polymer-based micelles, thus stopping the excitonic interaction and increasing the ^1^O_2_ generation. More importantly, we found a further increase of ^1^O_2_ generation at pH 5.0 in the presence of Tween-20 (**Figure 4**), which supported our hypothesis that the restoration of the ^1^O_2_ generation in the tumor tissue would be based on the disassembly of the micellar structures below the critical micellar concentration and the subsequent release of dPyF from the polymer conjugate and/or embedding into membranes in the tumorous tissue. In summary, the present **P‑hyd-dPyF** conjugate had to be safe and non-effective during the delivery, showing no or negligible activity, and the final therapeutic activity could be restored because of the micellar disruption and/or the release of freely active dPyF. Just after dissolution of the **P‑hyd-dPyF,** the quantum yield of singlet oxygen (∆φ) in the presence of surfactant (0.1% SDS) reached for all studied pH (5.0, 6.5, 7.4) value 0.5. This is highly comparable with free PyF, which shoved value of 0.52. Importantly, the **P-amide-PyF** had the value much lower (0.31), thus showing the benefit of the structure of novel **P‑hyd-dPyF** conjugate**.**

### In vitro pyropheophorbide-a derivative release from polymer conjugate

**Figure 5:**
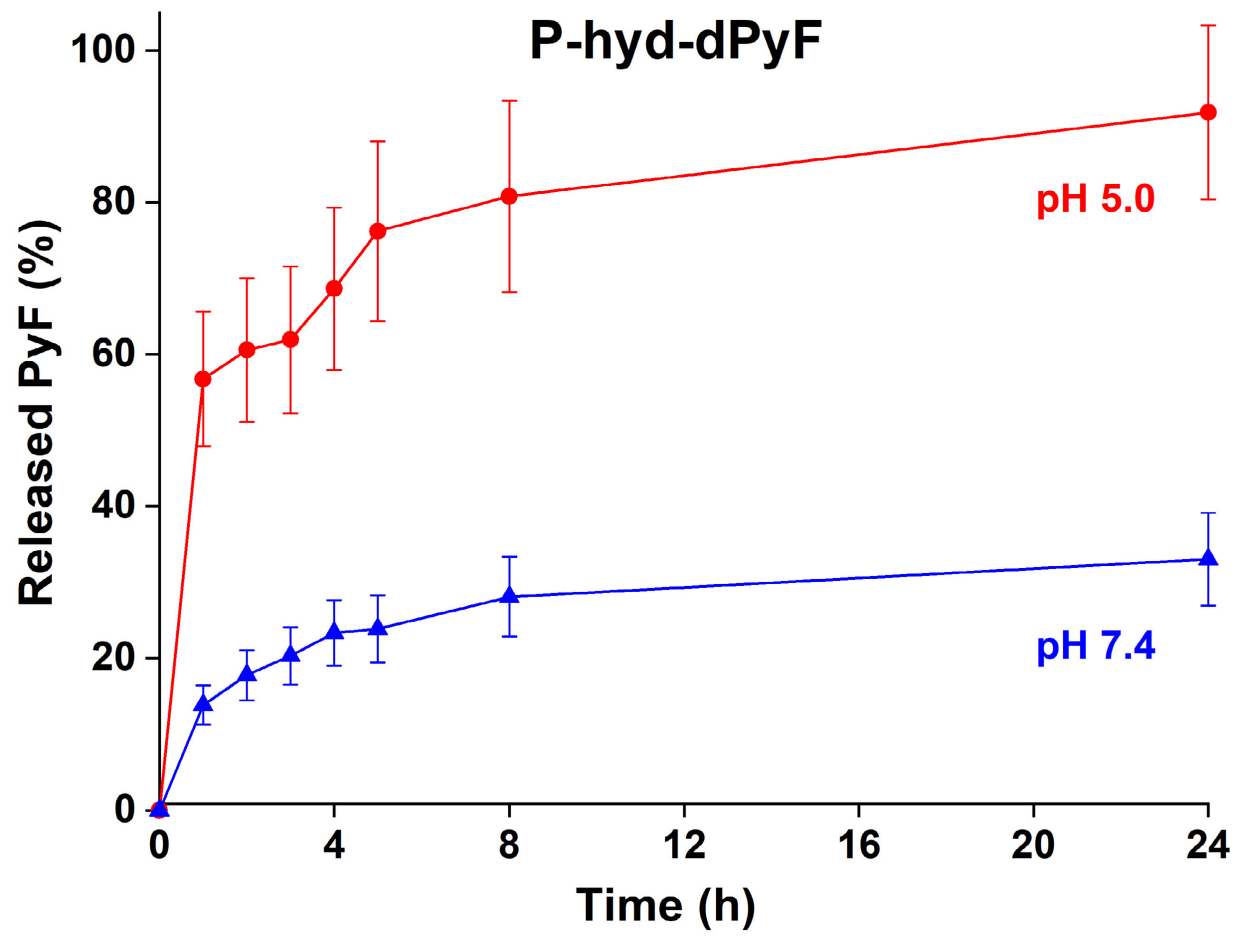
*In vitro* dPyF release from the polymer conjugate **P-hyd-dPyF**. Samples were incubated in 0.1M phosphate buffer solution with 0.05M NaCl at pH values of 5.0 and 7.4 at 37°C. Data represent mean ± SD.

The dPyF release from the conjugate **P-hyd-dPyF** was evaluated at 37°C in pH 5.0 and 7.4, modeling the acidic lysosome environment of the tumor cells, where the conjugates should be located after the cellular uptake, and the typical neutral blood conditions, respectively. The pH-dependent drug release was enabled by attaching dPyF to the polymer carrier via hydrazone bonds, which were quickly hydrolyzed at pH = 5.0, releasing up to 90% of dPyF after 24 h, in comparison with 30% of dPyF released at pH = 7.4 (**Figure 5**). The conjugate fulfills the basic criteria for a pH-sensitive pro-drug, relatively stable in the blood circulation and the fast dPyF release in the tumor tissue. The P-amide-Pyf conjugate was also incubated in both the buffers and no release of Pyf within 5 days was observed.

### Intracellular uptake of polymer conjugates in cultured cancer cells and spheroids

The above-described results showed the nano-micellar formulation of **P-hyd-dPyF**, and suggest its superior physiochemical characteristics, particularly the pH-responsive release profile compared with **P-amide-PyF**. We thus anticipated the superior PDT effect of **P-hyd-dPyF** and first examined its intracellular uptake dynamics by using cultured colon cancer C26 cells as well as the three-dimensional cell culture models, i.e., spheroids.

**Figure 6:**
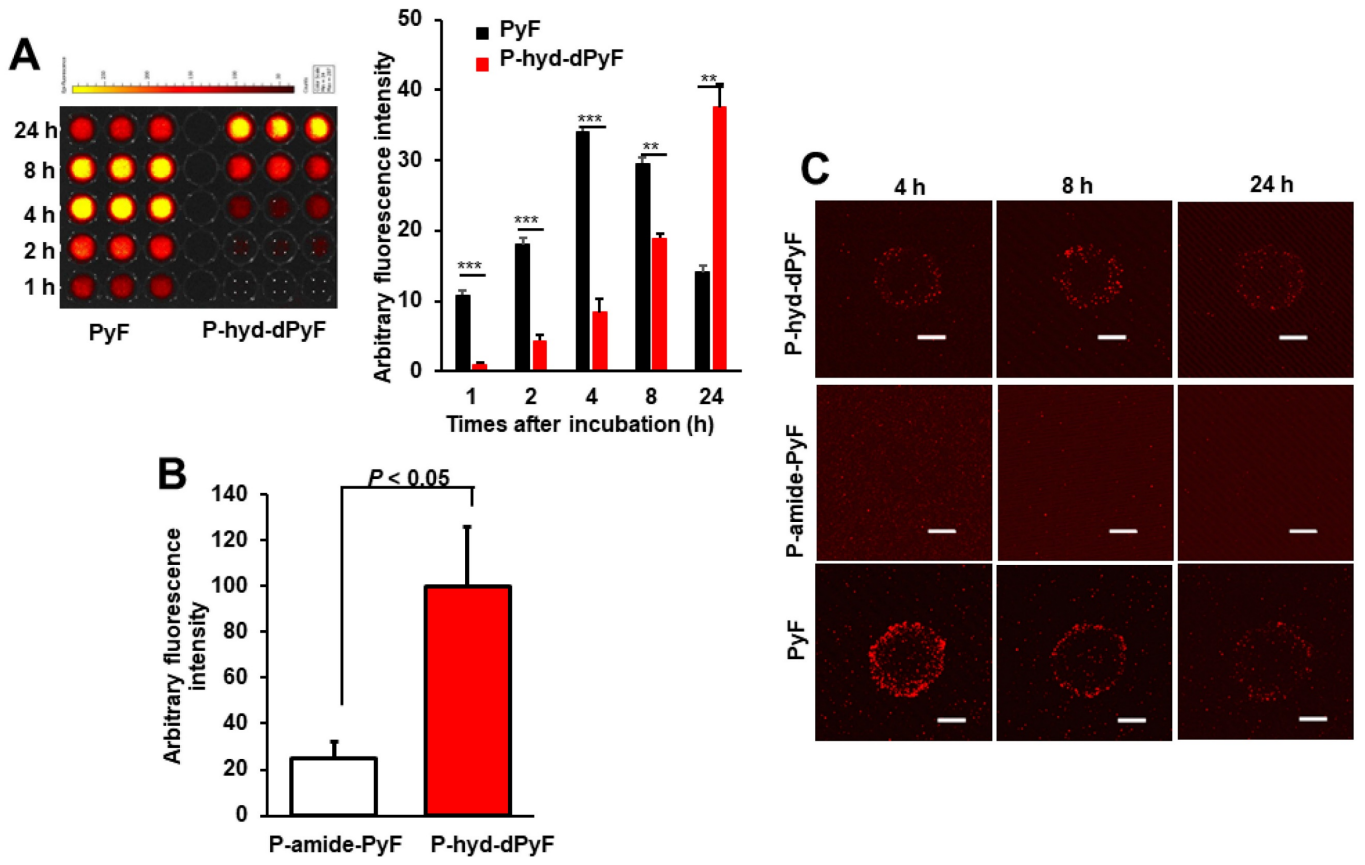
Intracellular uptake of polymer conjugates **P-hyd-dPyF** and **P-amide-PyF** compared with that of free PyF in cultured C26 colon cancer cells **(A, B)** and 3D spheroids **(C)**. **, *p* < 0.01; *** *p* < 0.001. Scale bars = 100 μm. See text for details. Data represent mean ± SD, n = 3.

Cultured C26 cells were treated with **P-hyd-dPyF** or free PyF, and the internalized drugs were visualized by detecting the fluorescence originated from PyF and semi-quantified by using the fluorescence intensity. **P-hyd-dPyF** showed a time-dependent intracellular uptake as the increase in the internalized drug continued up to at least 24 h (**Figure 6A**). In comparison, free PyF showed a similar but considerably faster intracellular uptake as the fluorescence originated from PyF reached a peak at 4-8 h after addition to the cells followed by the decrease in the fluorescence, which may suggest the degradation or beginning of the excitonic interaction due to the high local concentration 72 of PyF in the cells (**Figure 6A**). More importantly, compared with **P-amide-PyF**, **P-hyd-dPyF** exhibited a significantly higher intracellular uptake, which was evaluated after 2 h of treatment and quantified by fluorescence (**Figure 6B**). Here, we assumed that there was a concurrence of the internalization of dPyF released outside the tumor cells and **P-hyd-dPyF** itself. These findings suggest that the release of free dPyF is an important step for its efficient cell internalization, thus fulfilling the therapeutic effect. Moreover, the internalization of dPyF using **P-hyd-dPyF** was comparable to free PyF, showing the huge advantage of such stimuli-responsive nanomedicine already *in vitro*. We also expected the slower intracellular uptake profile of **P-hyd-dPyF** than that of free PyF as the dPyF release from the polymer conjugate is a time-dependent event. We further examined the behavior of polymer conjugates versus that of free PyF by using an *in vitro* 3D C26 spheroid model and confocal microscopy (**Figure 6C**). The uptake of free PyF by spheroids was clearly observed 4 h after the treatment and then gradually decreased similarly, as shown in the cultured C26 cells (**Figures 6A and C**). In parallel with the results shown in Figure 6A, **P-hyd-dPyF** exhibited a comparable but slower uptake than free PyF, whereas no visible fluorescence appeared up to 8 h after the **P-amide-PyF** treatment (**Figure 6C**). These findings reveal the superior uptake property of **P-hyd-dPyF** to that of **P-amide-PyF**. We hypothesized that the beneficial uptake of **P-hyd-dPyF** could be linked to the enhancement of the therapeutic effect. Importantly, Figure 6B indicates that micellar polymer conjugates could be taken up by cancer cells and spheroids; however, besides the small amount, the fluorescence quenching in the micellar formation resulted in the invisibility of the fluorescence in the cells and spheroids (**Figure 6C**).

### In vitro PDT effect of polymer conjugates

To investigate the PDT effect of **P-hyd-dPyF**, we first evaluated its cytotoxicity in the cultured C26 colon cells in the dark or under illumination. For the assay of dark cytotoxicity, the cells were treated for 48 h in the dark, and for PDT, after 24 h of treatment in the dark, illumination using a blue light source with the maximum absorption of 420 nm was carried out (1 J cm^-2^), followed by further 24 h incubation in the dark. In our previous study using P-amide-PyF, we found red light irradiation (680 nm) showed neglectable PDT effect compared to blue light exposure, suggesting the light corresponding to the maximum absorbance of PyF is most suitable for PDT using PyF 6. Accordingly, irradiation using blue light was carried out in this study.

**Figure 7:**
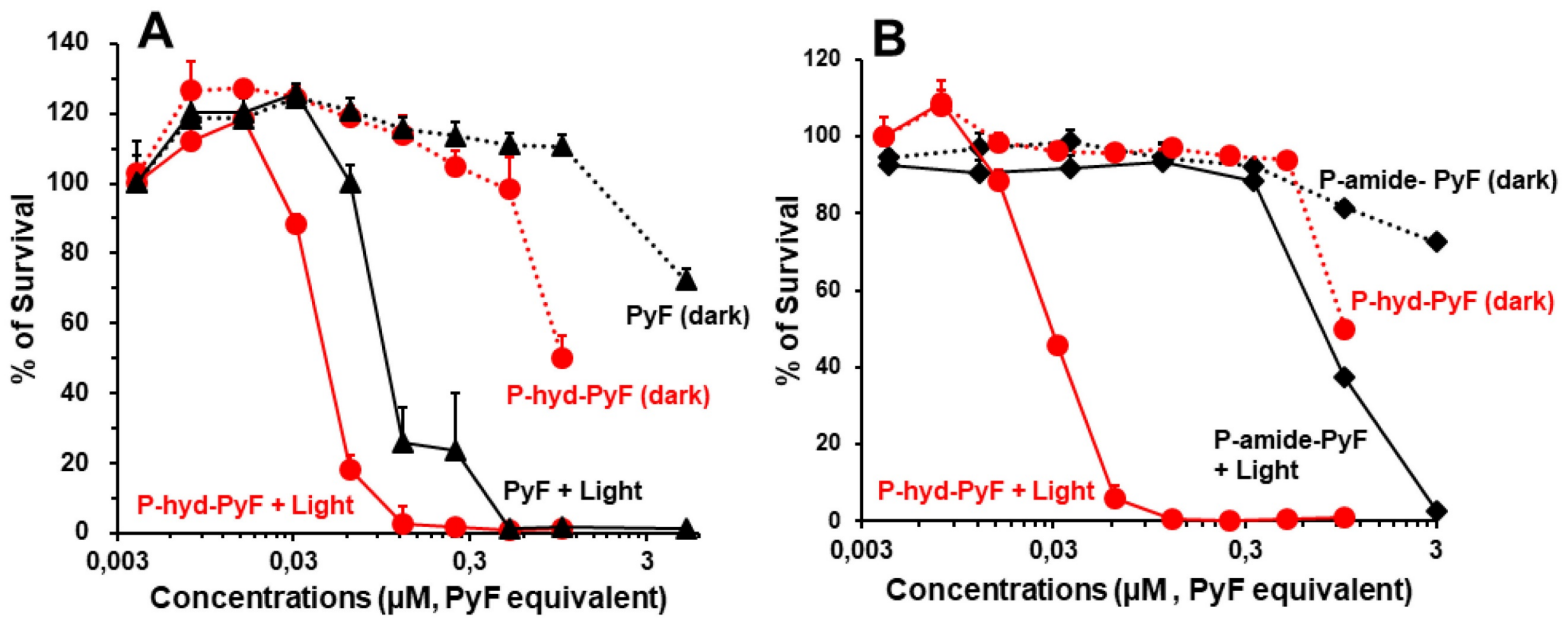
*In vitro* cytotoxicity and PDT effect of polymer conjugates compared with that of free PyF. Cell viability of C26 colon cancer cells as examined using MTT assay: **(A) P-hyd-dPyF** *vs.* free PyF. **(B) P-hyd-dPyF** *vs.* **P-amide-PyF**. See text for details. Data represent mean ± SD, n = 6-8.

The cell viability was examined using MTT assay. In the dark, free PyF and **P-hyd-dPyF** showed no apparent cytotoxicity up to 0.5 μmol in the medium. Instead, after illumination using the blue light source, their cytotoxicity remarkably increased in a dose-dependent manner: all of the cells died at 0.1 μmol. More interestingly, the PDT effect of **P-hyd-dPyF** was comparable and even stronger than that of the free PyF (**Figure 7A**), probably because of the accumulative internalization profile of **P-hyd-dPyF**: the intracellular uptake of **P-hyd-dPyF** increased time-dependently to at least 24 h, whereas the internalization of free PyF reached the peak at 4-8 h after which it decreased (**Figure 6A**). Moreover, we evaluated the conjugates' cytotoxicity and observed a considerably stronger PDT effect of **P-hyd-dPyF** (IC_50_ of approximately 0.03 μmol) than that of **P-amide-PyF** (IC_50_ of approximately 0.6 μmol) (**Figure 7B**). These findings again support that the internalization and rapid release of free dPyF plays a critical role in the PDT effect of PyF conjugates: **P-hyd-dPyF** thus exhibited a superior PDT effect to that of **P-amide-PyF** because of the cleavable hydrazone bond and the preferable release profile (**Figure 5**).

To further confirm the PDT effect of **P-hyd-PyF**, we conducted measurements of intracellular ROS generation during PDT using **P-hyd-PyF**. The results revealed a dose-dependent increase in intracellular ROS levels (**Figure S3.1**). These findings provide additional support for the notion that the cytotoxicity of **P-hyd-PyF** is attributed to the PDT effect, specifically the generation of ROS.

### Tissue distribution of P-hyd-dPyF and in vivo imaging

**Figure 8:**
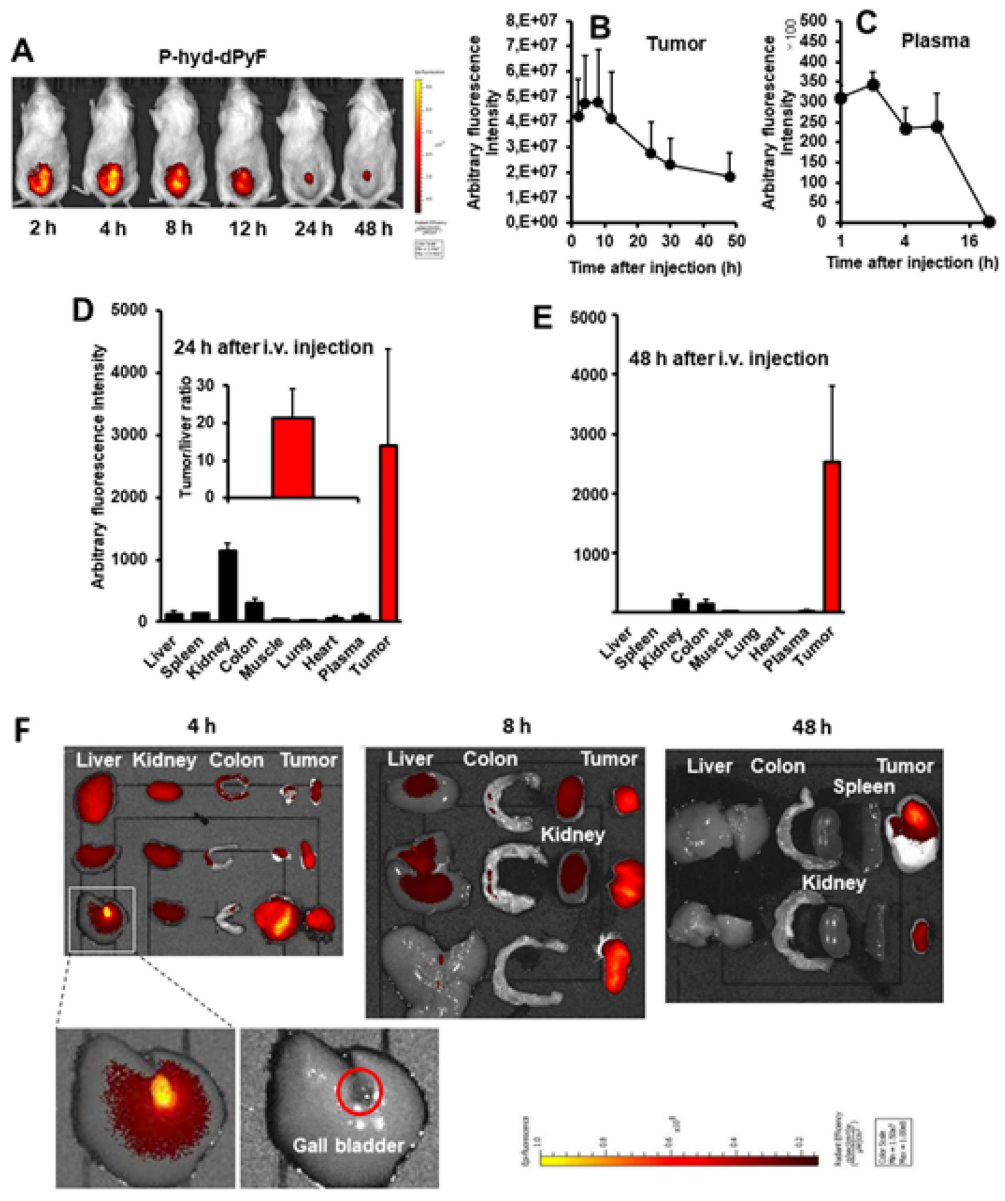
Tissue distribution of **P-hyd-dPyF** after intravenous injection (5 mg kg^-1^, PyF equivalent) using a mouse sarcoma S180 solid tumor model. **(A)*** In vivo* imaging. **(B)** Semi-quantification of PyF accumulated in the tumor. **(C)** Plasma pharmacokinetics. **(D)** Tissue distribution of **P-hyd-dPyF** at 24 h after intravenous injection (inset shows the ratio of drug in the tumor *vs.* in the liver). **(E)** Tissue distribution of **P-hyd-dPyF** at 48 h after intravenous injection. **(F)** *Ex vivo* imaging. See text for details. Data represent mean ± SD, n = 6-8.

One concern regarding **P-hyd-dPyF** is how the pH-stimuli sensitiveness would affect the *in vivo* behavior. We thus investigated the *in vivo* tissue distribution dynamics of **P-hyd-dPyF** by injecting the conjugate at 5 mg kg^-1^ (PyF equivalent) in S180 tumor-bearing mice when the tumor grew to approximately 10 mm in diameter. Figures 8A and 8B show that the *in vivo* imaging exhibited an extensive fluorescence in the tumor with no or very low background fluorescence, which correlated with the semi-quantification of the drug accumulated in the tumor. Moreover, we observed a strong fluorescence at 2 h after the intravenous injection, and these high levels were maintained in the tumor to at least 12 h, and then gradually decreased; however, even at 48 h after the intravenous injection, a comparable strong fluorescence signal could still be detected in the tumor (**Figures 8A and B**). In parallel, **P-hyd-dPyF** maintained a relatively high concentration in the plasma, with the plasma half-life > 12 h (**Figure 8C**). These results clearly reveal that **P-hyd-dPyF** behaves as a nanomedicine *in vivo* showing a prolonged circulation time and tumor selective accumulation by taking advantage of the EPR effect.

After the mice were sacrificed, tumors and normal tissues were collected, and dPyF was extracted to measure fluorescence spectroscopy. When we examined the distribution of **P-hyd-dPyF** in different tissues, we found a high plasma concentration along with a high drug accumulation in the tumor up to 8 h after the intravenous injection. Moreover, comparable levels of the drug were found in many major organs (e.g., the liver, spleen, kidney, and colon) (**Figures 8F and S3.2**). However, more importantly, at 24 h after the intravenous injection, the amount of drug in the normal tissues considerably decreased, whereas a high level in the tumor was maintained, resulting in a tumor/liver drug ratio of 20; i.e., the released** dPyF** in the tumor was 20 times higher than that in the liver (**Figure 8D**). Notably, almost no drug was found in the normal tissues after 48 h, whereas a high level in the tumor still remained (**Figure 8E**). We also observed similar results for the fluorescence imaging of dissected tissues (**Figure 8F**). Importantly, the tissue distribution for **P-hyd-dPyF** was completely different in comparison to **P-amide-PyF** 6 showing more favorable tumor accumulation. The **P-amide-PyF** was after 24 h predominantly localized in the liver and to lesser extent in spleen, plasma and tumor. We summarize that the stimuli-sensitive **P-hyd-dPyF** enable to enhanced the PyF biodistribution with predominant tumor accumulation and low off-target localization.

**Figure 9:**
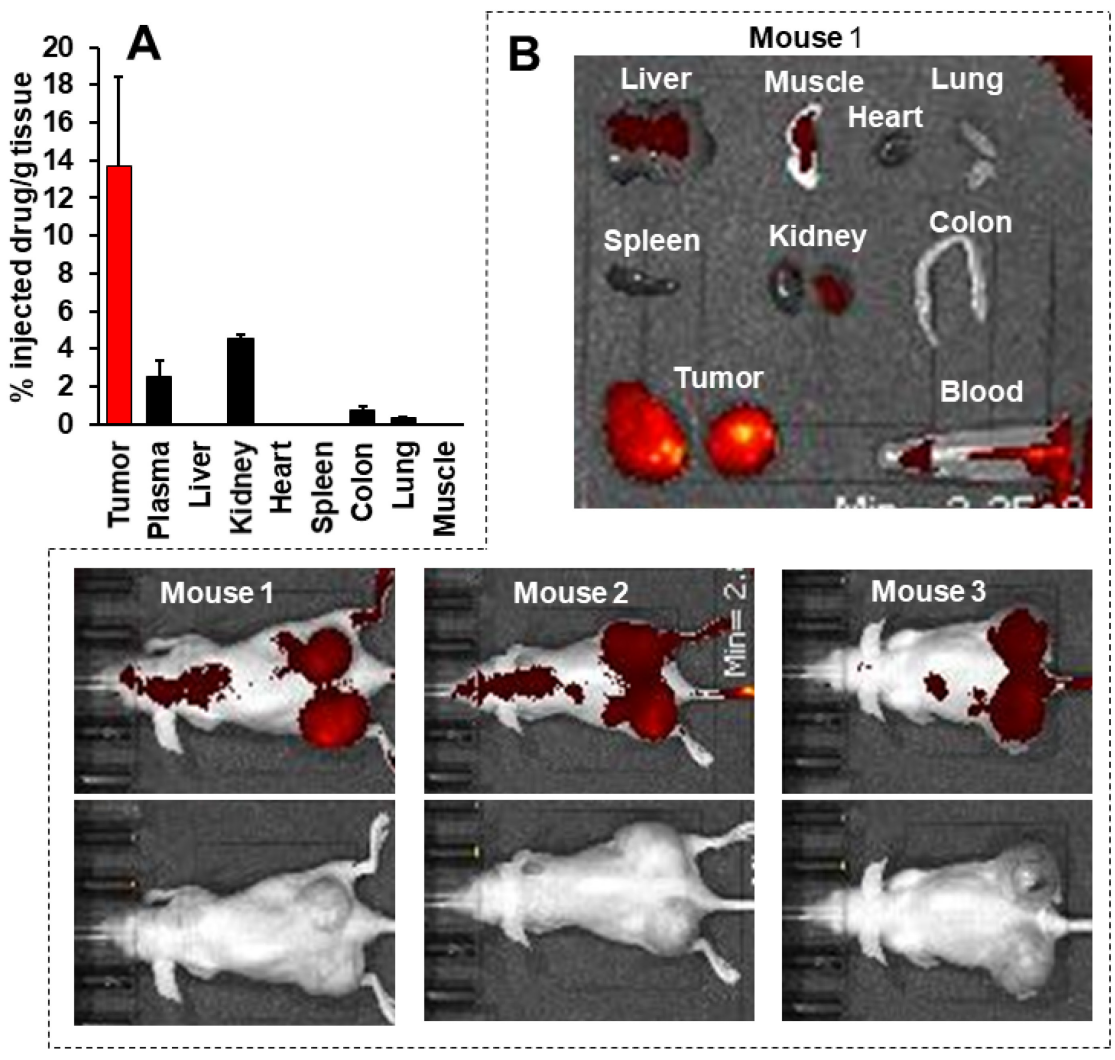
Tissue distribution of **P-hyd-dPyF** after intravenous injection (5 mg kg^-1^, PyF equivalent) in human ovarian cancer xenograft. **(A)** PyF in different tissues was quantified using the standard curve of **P-hyd-dPyF** and fluorescence spectroscopy. **(B)*** In vivo* (upper, fluorescence image; lower, black/white image) and* ex vivo* imaging. See text for details. Data represent mean ± SD, n = 6-8.

This body distribution profile of **P-hyd-dPyF** was confirmed by using a human ovarian cancer xenograft model (**Figures 9A and B**), and the quantification analysis showed that the tumor concentration of **P-hyd-dPyF** was approximately 14% of the injected drug/g tissue (**Figure 9A**). These findings indicated the beneficial tissue distribution dynamics of **P-hyd-dPyF**, which not only exhibited a sustained and targeted accumulation in the liver but also could achieve an extremely high tumor/liver ratio that is hardly seen in many other nanomedicines, particularly nano photosensitizers. In fact, these nanomedicines could be taken up by the reticuloendothelial system in the liver and spleen, 72, 73 and our previously developed HPMA polymer conjugated to photosensitizers (i.e., HPMA-zinc protoporphyrin and P-amide-PyF) already showed a relatively high liver accumulation.^6^, ^48^ We considered that the low distribution in the liver or clearance from the liver and other normal tissues was mostly because of the release of dPyF from **P-hyd-dPyF** as the released dPyF was excreted from urine or from bile after metabolization in the liver. This notion was supported by the *in vivo* distribution profile of **P-hyd-dPyF**, which showed relative high levels of the drug in the kidney even after 24 h (**Figures 8D and 9A**), and the fluorescence in the liver at 4 h after the intravenous injection was found mostly in the gall bladder (**Figure 8F**). However, polymer conjugates and the released highly hydrophobic dPyF could not be cleared from the tumor efficiently because of the poor lymphatic function; 74, 75 therefore, they were retained in the tumor for a prolonged time. Moreover, because of the pH-responsive release property of **P-hyd-dPyF**, a rapid release of dPyF could be attained in the weak acidic condition of solid tumors, 76 which would subsequently lead to the increased intracellular uptake, resulting in an efficient PDT effect.

The *in vivo* imaging of **P-hyd-dPyF** showed a clear fluorescence signal in the tumor with very low contrast background signal (**Figures 8A and F**). One important advantage of PyF is that it can be excited using the light at 670 nm in which the long wavelength ensures the deep penetration into the tissues and that it can emit fluorescence with a wavelength longer than 700 nm that facilitates the detection with less interference of autofluorescence. Using this Ex/Em combination, we detected the tumor with high selectivity and specificity as supported by the results shown in Figures 8A and 8F. In this context, we found out that the selection of an appropriate wavelength is a key issue, as this choice allows a strong and selective tumor fluorescence with almost no background. We observed that imaging experiments had to be performed at λ_exc_ = 670 nm that served only for diagnosis, because no ^1^O_2_ was produced together with a high contrast of the tumor tissue (lower fluorescence of the polymer conjugate and higher fluorescence of released dPyF). Instead, in our experiments, illumination at λ_exc_ = 420 nm reached a considerably better therapeutic effect as a result of the ^1^O_2_ generation, even though the penetration depth of blue light is very limited. Based on previous results, 77 one might speculate that the reason for the wavelength dependency of the therapeutic effect might be the different penetration depth and the corresponding excitation intensity inside the tumor.

Moreover, we are aware that the tumor location is important as PDT can only treat tumors where light can reach. Besides superficial tumors with easy access to light, it is possible to apply interstitial, endoscopic, or laparoscopic devices for the delivery of light into more difficult access treatment sites. Unfortunately, solo therapy using PDT cannot be applied to treat metastatic tumors or patients with allergies to these compounds 8, 9. It is thus important and of great necessity to develop a strategy to detect and differentiate tumors from normal tissues and then carry out PDT pin-point to the tumor. The results in this study suggest that **P-hyd-dPyF** may become a potential candidate drug for this therapeutic approach because of its high tumor selectivity and superior *in vivo* imaging property, which warrants further investigation.

### In vivo therapeutic effect (PDT) of polymer conjugates

Finally, we investigated the *in vivo* PDT effect of **P-hyd-dPyF** by using the mouse sarcoma S180 solid tumor model. Because of the highly tumor selective accumulation demonstrated above, we expected the superior therapeutic effect of **P-hyd-dPyF**. According to the tissue dynamics of **P-hyd-dPyF** (**Figure 8**), we carried out the illumination by using a xenon light source for 5 min at 24 h and 48 h after the conjugates' administration; when **P-hyd-dPyF** was delivered and retained in the tumor, whereas no or very few remained in normal tissues. Moreover, this time setting ensured the dPyF release that further increased the PDT efficacy. The illumination was carried out with a broad wavelength light of 400-700 nm, which could ensure sufficient illumination intensity while simultaneously covering the appropriate light range (i.e., 420 nm) to efficiently excite PyF. Consequently, this strategy enhanced the therapeutic outcome of cancer treatment when compared with the conventional chemotherapy.

**Figure 10:**
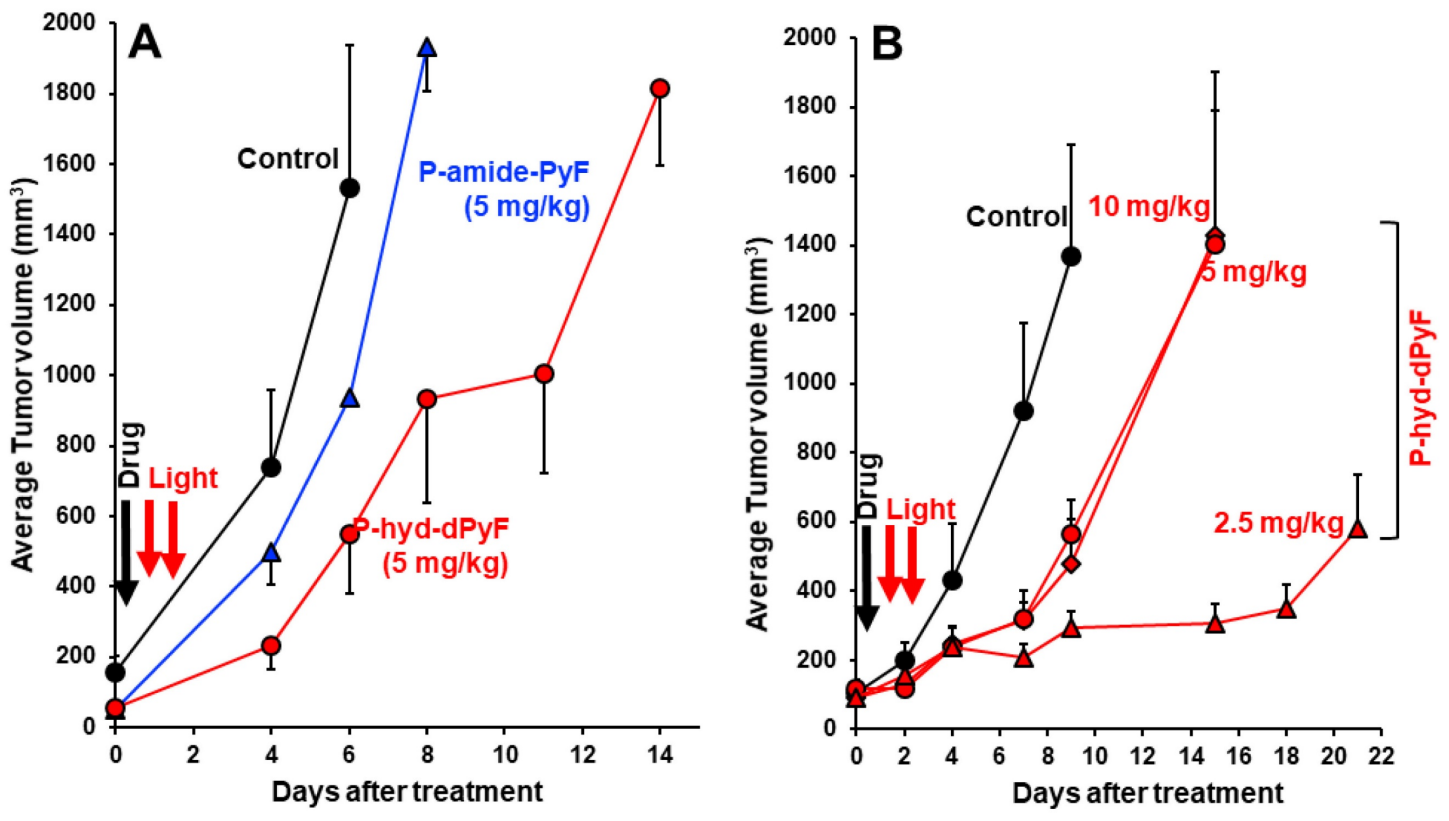
*In vivo* PDT effect of **P-hyd-dPyF** in mouse sarcoma S180 solid tumor model. **(A) P-hyd-dPyF** *vs.* **P-amide-PyF** (both at 5 mg kg^-1^, PyF equivalent). **(B)** Influence of different doses of **P-hyd-dPyF** on the PDT effect. See text for details. Data represent mean ± SD, n = 6-8.

As expected, we achieved a significant tumor growth suppression by one round of PDT using **P-hyd-dPyF** at 5 mg kg^-1^ (PyF equivalent), and the effect was considerably superior to that of the PDT using **P-amide-PyF,** the tumor inhibitory rate of** P-hyd-dPyF** and **P-amide-PyF** at day 6 after treatment was 64.2% and 39.1%, respectively (**Figure 10A**). However, when we further investigated the optimal dosing and therapeutic regimen, unexpectedly and interestingly, we found that the lower dose of **P-hyd-dPyF** (i.e., 2.5 mg kg^-1^, PyF equivalent, tumor inhibitory rate of 78.2% at day 9 after treatment) showed a considerably better therapeutic effect than the higher doses (i.e., 5 mg kg^-1^ and 10 mg kg^-1^, PyF equivalent, tumor inhibitory rate of 58.7% and 65.0%, respectively, at day 9 after treatment), and almost a complete inhibition of tumor growth was observed up to 18 days after the treatment (**Figure 10B**). Similarly, the two-round PDT using 5 mg kg^-1^ of **P-hyd-dPyF** exhibited similar results to those of the one-round PDT of 10 mg kg^-1^ **P-hyd-dPyF**, both of which showed a decreased therapeutic effect than that of the one-round of 5 mg kg^-1^ **P-hyd-dPyF** (**Figure S.3.3A**). Although it is not completely clear why **P-hyd-dPyF** exhibited the reversed dose-dependency, potential explanations include the following: i) the relatively low dose of 2.5 mg kg^-1^ was sufficient to exert a sufficient PDT effect, mostly because of the tumor selective accumulation and retention of **P-hyd-dPyF**, and ii) higher doses of **P-hyd-dPyF** were not preferable for PDT. Regarding the later issue, a recent study from our group indicated that very intense illumination during PDT negatively influenced the ^1^O_2_ generation and the PDT effect, in which photosensitization and oxygen consumption exceeded the oxygen supply, limiting the generation of ^1^O_2_ to blood vessels and their direct vicinity, whereas less ^1^O_2_ was generated in the surrounding tumor tissues 78. Similar events might happen in the case of the higher doses of **P-hyd-dPyF**, which triggered a rapid and vast generation of ^1^O_2_ mostly in blood vessels but without the sufficient PDT effect in the tumor tissues because of the lack of oxygen. Moreover, ^1^O_2_ and ROS generated in the blood vessels would destroy them, further exacerbating the hypoxic or anoxic condition, consequently influencing the PDT effect. It is thus reasonable to use a relative low dose of **P-hyd-dPyF** and moderate intensity of light as the optimal PDT regimen. A future follow-up study will focus on this issue to clarify the influence of dosing/illumination balance on the PDT effect as well as the fluorescence imaging efficacy, towards the clinical development of **P-hyd-dPyF** as a theranostic agent.

To verify the beneficial therapeutic effect of **P-hyd-dPyF** the *in vivo* PDT effect of both polymer conjugates was evaluated by using the mouse colon cancer C26 solid tumor model, see **Figure S3.4**. Importantly, we have found similar superior treatment efficacy of **P-hyd-dPyF** as in the case of mouse sarcoma model. Also, the reverse dose dependency was observed showing excellent treatment efficacy of the stimuli sensitive theranostics **P-hyd-dPyF.** In addition, during the PDT treatment using **P-hyd-dPyF** as described above, no apparent body weight loss (**Figures S3.3B, S3.3C, and S3.5A**) and no apparent inflammatory changes (reddish and blackish coloration) in the skin were observed, not only in the skin away from the tumor but also in the skin surrounding the tumor, which was illuminated during the treatment. Histological examination (H&E) staining also showed no apparent histological changes in the major organs, i.e., the liver and kidney, induced by the treatment (**Figure S3.5B**). These findings suggest the safety of the **P-hyd-dPyF-**based PDT.

### Singlet oxygen and PyF phosphorescence in vivo

**Figure 11:**
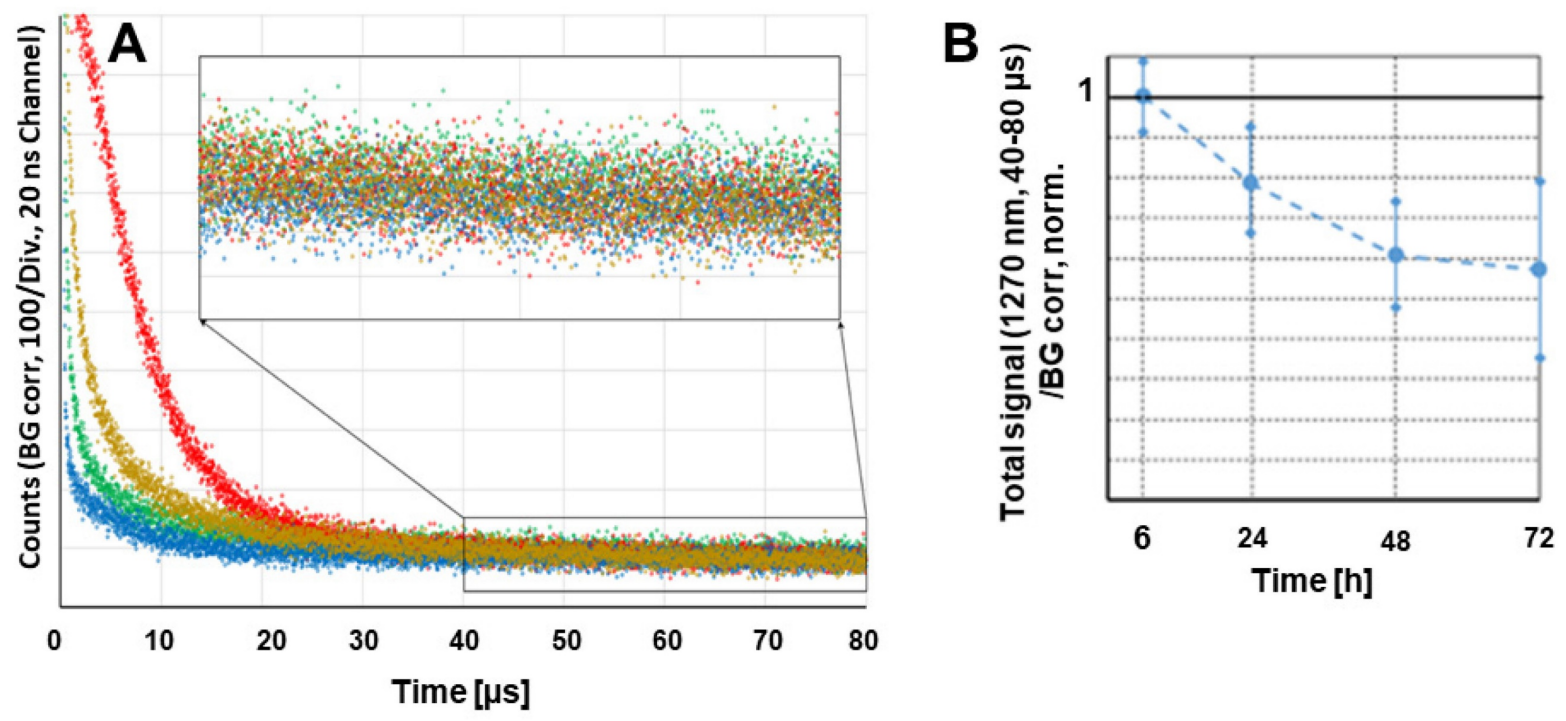
Time-resolved phosphorescence around 1270 nm detected in four different tumors, 6 h after the injection of **P-hyd-dPyF**, Insert: Bigger representation of the marked diagram area **(A)**. Cumulative counts for the detection around 1270 nm in the time window of 40-80 µs after excitation at different time points after the systemic injection of **P-hyd-dPyF**, normalized to the value of **P-hyd-dPyF** after 6 h **(B)**.

We tested whether the accumulation of the polymer conjugate or better, the accumulation of active dPyF, could be optically quantified *in vivo* without harming the animal. Such a method would allow considerably better supervision while reducing the number of required animals. The effect that had to be exploited for this quantification in the tumor tissue, was the PDT-induced anoxia, which was described previously 77, 78. The PDT treatment intrinsically consumes oxygen, because of the chemical reactions between the generated singlet oxygen and target cell components, mainly proteins. This resulted in very slow decaying phosphorescence signals, originating from the extravasated PyF in the tumor tissue, already at considerably low illumination intensities. We used around 60 mW cm^-2^, which was clearly above the estimated threshold for this effect to occur. Figure 11A shows the phosphorescence kinetics of the four investigated tumors 6 h after the intravenous injection of **P-hyd-dPyF**. The optical fiber was positioned centrally on the tumor with direct skin contact. It was quite obvious that there were huge differences for the investigated tumors in the time region of 0 to 30 µs after excitation. Following our previous explanation, we assigned these signal components to the singlet oxygen that was generated by such a drug that was still circulating in the blood vessels. In the blood flow, there was no lack of oxygen and the signal components showed the typical double exponential behavior, masked by the strong short-term artifact of such measurements. Such signals could be detected for days all over the body of the mouse after the drug injection. However, the intensity varied considerably with the location, which was no surprise as the number of blood vessels and their size varied as well. This also applied, of course, to blood vessels that were near or in the tumor, which contributed to the detected signal. In principle, such direct observation of the generated singlet oxygen would be an option to quantify the drug concentration in blood, if we managed to assure the better reproducibility of the positioning. The point here, however, was that the signals from the dPyF in the blood decayed a few tens of microseconds after excitation, while in the time window of 40-80 µs, we mainly detected the phosphorescence of the extravasated drug. For tumors of similar size (5-8 mm) and identical applied drug amounts, this signal component showed little variation. As we expected immediate anoxia in the tumor after illumination, the PyF phosphorescence intensity had to be, in the first approximation, proportional to the local PyF concentration in the tissue. This measurement was considerably robust against the misplacement of the fiber tip, as the tumors were bigger than the optical fiber and because the signal scattering was average over several cubic millimeters of the tumor.

The measurements were repeated at several time points after injection. Figure 11B shows the average values and error margins of the integrated signal counts within the time window of 40-80 µs after excitation, for each of these moments. The relative margin of the signals 6 h after the injection was just approximately 10%. For later times, the margin increased, but the development of the signal intensity was obvious. The signal intensity for **P-hyd-dPyF** was the highest just 6 h after injection and decayed slowly. This corresponds to our other results and demonstrates that time-resolved phosphorescence detection is an appropriate method to determine the amount of active dPyF in the tumor.

## Conclusions

Herein, we successfully developed and deeply evaluated polymer-based nanomedicines serving as stimuli-responsive theranostics in the treatment and visualization of solid tumors. PyF bound by the pH-sensitive hydrolyzable hydrazone bond to the polymer carrier enabled the tumor site-associated simultaneous PDT and tumor imaging. The safety of such theranostics in circulation was ensured by their self-assembly into micellar structures in aqueous solutions. Moreover, the stimulus-sensitivity behavior showed beneficial properties for the tumor-targeted delivery, reducing their accumulation in the liver and spleen. The nanomedicine was stable during the transport to the tumor tissue, and after the micelles' selective accumulation in solid tumors via the EPR effect, their disruption was confirmed by the appearance of a strong fluorescence signal with almost no background at λ_exc_ = 670 nm. The beneficial tissue distribution dynamics of the tumor microenvironment-responsive system also resulted in a superior PDT effect because of the specific PyF release and activation of its theranostic nature in the acidic tumor environment. Once in the tumor, illumination at λ_exc_ = 420 nm allowed a remarkable antitumor PDT effect in the mouse sarcoma S180 solid tumor model. Indeed, low doses of PyF proved to be optimal for superior therapeutic effects that resulted from the ^1^O_2_ generation. We are convinced that such theranostic that is functional at low doses is an excellent candidate for further preclinical and clinical development.

## Supplementary Material

Supplementary figures.

## Figures and Tables

**Table 1:** Physico-chemical characterization of polymer precursors.

Polymer precursor	Functional group	Content of functional group (mol.%)^a^	*M*_n_ (g mol^-1^)^b^	*M*_w_ (g mol^-1^)^b^	*Ð* ^b^	*D*_H_ ± SD (nm)^c^
**P1**	hydrazide	8.6	25,500	26,400	1.03	8.7 ± 0.4
**P2**	amine	7.0	29,200	30,200	1.03	9.9 ± .0.5

^a^ Molar contents of hydrazide or amine groups were determined by UV-Vis spectrophotometry using the 2,4,6-trinitrobenzene-1-sulfonic acid (TNBSA) assay method. ^b^ The number-average molecular weight (*M*_n_), weight-average molecular weight (*M*_w_), and dispersity (*Ð*) of the polymer precursors were determined by size-exclusion chromatography (SEC) using multiangle light scattering (MALS) and refractive index (RI) detectors. ^c^ Hydrodynamic diameters (*D*_H_) were evaluated in PBS (pH 7.4) at 3 mg mL^-1^ by using a Nano-ZS instrument, Malvern, with the laser at λ = 632.8 nm.

**Table 2:** Physicochemical characterization of polymer conjugates.

Polymer conjugates	Prepared from derivative (spacer)	Content of PyF in wt.% (molecules of PyF per polymer chain)^a^	*D*_H_ ± SD (nm)^b^
**P-hyd-dPyF**	5-hydroxy-2-pentanone	6.6 (3)	15.9 ± 4.3
**P-amide-PyF**	Pentafluorphenyl ester of PyF	4.8 (3)	14.8 ± 2.4

^a^ The amount of PyF in the conjugates was determined by UV-Vis spectrophotometry in methanol by using λ_max_ = 416 nm and λ_max_ = 668 nm. ^b^ Hydrodynamic diameters (*D*_H_) were evaluated in PBS (pH 7.4) by using a Malvern Ultra equipped with a fluorescence filter (laser at λ = 632.8 nm).

## Abbreviations

AIBN: 2,2′-azo*bis*isobutyronitrile
APMA-Boc: *N*-(3-*tert*-butoxycarbonyl-aminopropyl)methacrylamide
CTA: chain transfer agent
DCM: dichloromethane
DLS: dynamic light scattering
DMA: *N,N*-dimethylacetamide
DMAP: 4-(dimethylamino)pyridine
DMF: *N,N*-dimethylformamide
DMSO: dimethyl sulfoxide
dPyF: pyropheophorbide-a 5-hydroxy-2-pentanone ester
EDC: *N*-ethyl-*N*′-(3-dimethylaminopropyl)carbodiimide hydrochloride
EPR: enhanced permeability and retention effect
ESR: electron spin resonance spectroscopy
HPLC: high-performance liquid chromatography
NIR: near infrared
PBS: phosphate buffered saline
PDT: photodynamic therapy
pHPMA: *N*-(2-hydroxypropyl)methacrylamide copolymer
PS: photosensitizer
PTX: paclitaxel
PyF: pyropheophorbide-a
ROS: reactive oxygen species
SDS: sodium dodecyl sulfate
TLC: thin layer chromatography
TNBSA: 4,6-trinitrobenzene-1-sulfonic acid
V-70: 2,2ʼ-azo*bis*(4-methoxy-2,4-dimethylvaleronitrile
4-oxo-TEMP: 4-oxo-2,2,6,6-tetramethyl-1-piperidinyloxy, free radical
