# Supplementary material for: Polymer theranostics with multiple stimuli-based activation of photodynamic therapy and tumor imaging
Source: Theranostics. 2023 Sep 4;13(14):4952–73. doi: 10.7150/thno.86211 (PMC10526675; doi:10.7150/thno.86211)
Supplement: Supplementary file 1 — Supplementary figures. [file thnov13p4952s1.pdf]

Electronic Supplementary Information

Polymer theranostics with multiple stimuli-based activation of photodynamic therapy and tumor imaging

Marina Rodrigues Tavares[1#], Rayhanul Islam[2#], Vladimír Šubr[1], Steffen Hackbarth[3], Shanghui Gao[2], Kai Yang[2], Volodymyr Lobaz[1], Jun Fang[2*], Tomáš Etrych[1*]

1. Institute of Macromolecular Chemistry, Czech Academy of Sciences, Heyrovského nám. 2, 16200 Prague, Czech Republic

2. Laboratory of Microbiology and Oncology, Faculty of Pharmaceutical Sciences, Sojo University, Kumamoto 860-0082, Japan

3. Institute of Physics, Photobiophysics, Humboldt University of Berlin, Newtonstr. 15, 12489 Berlin, Germany

[*] Corresponding authors. E-mail address: etrych@imc.cas.cz, fangjun@ph.sojo-u.ac.jp.

# These authors contributed equally to this paper.

Table of content

1. Synthesis of monomers, chain transfer agent and polymer precursors

Figure S1. Synthesis of polymer precursors poly(HPMA-*co*-MA-AH-$NHNH_2$) (**P1**) and poly(HPMA-*co*-MA-APMA) (**P2**).

2. Physico-chemical characterization

2.1. Nuclear magnetic resonance spectra of monomers, chain transfer agent, polymer precursors and conjugates

Figure S2.1.1. ^{1}H NMR spectrum of HPMA

Figure S2.1.2. ^{1}H NMR spectrum of MA-AH-NHNH-Boc

Figure S2.1.3. ^{1}H NMR spectrum of S-2-cyano-2-propyl-S`-ethyl trithiocarbonate

Figure S2.1.4. ^{1}H NMR spectrum of derivative dPyF

Figure S2.1.5. ^{1}H NMR spectrum of precursor poly(HPMA-*co*-MA-AH-$NHNH_2$)

Figure S2.1.6. ^{1}H NMR spectrum of P-hyd-dPyF

Figure S2.1.7. ^{1}H NMR spectrum of precursor poly(HPMA-*co*-MA-AP-NH_2)

Figure S2.1.8. ^{1}H NMR spectrum of P-amide-PyF

2.2. Field flow fractionation and Size exclusion chromatography

Figure S2.2. Polymer precursor **P1** and polymer conjugate **P-hyd-dPyF**

2.3. UV–Vis spectrophotometry

Figure S2.3 UV-Vis spectra of pure PyF, derivative dPyF, conjugate **P-hyd-dPyF**, and conjugate **P-amide-PyF**

2.4. TEM microscopy

Figure S2.4 TEM microscopy of conjugate **P-hyd-dPyF**

2.5 Isothermal titration calorimetry

Figure S2.5 Heat of dilution of **P1**, **P2**, **P-amide-PyF** and **P-hyd-dPyF** polymers measured by ITC in PBS at 37°C

3. *In vitro* PDT effect, *in vivo* tissue distribution and PDT antitumor activity

Figure S3.1.

Figure S3.1. Tissue distribution of **P-hyd-dPyF**

Figure S3.2. *In vivo* PDT effect of **P-hyd-dPyF**

4. References

1. Synthesis of monomers and polymer precursors

N-(2-hydroxypropyl)methacrylamide (HPMA)

N-(2-hydroxypropyl)methacrylamide (HPMA) was synthesized by the reaction of methacryloyl chloride and 1-aminopropan-2-ol in dichloromethane in the presence of anhydrous sodium carbonate as described before [1]. Melting point: 69 °C. Elemental analysis: calculated C 58.72 %, H 9.15 %, N 9.78 %; Found C 58.98 %, H 9.18 %, N 9.82 %. ^{1}H NMR (DMSO-d_6, 600 MHz): δ = 7.80 ppm (s, 1H, N*H*); δ = 5.65 ppm (m, 1H, =CH_2); δ = 5.31 ppm (m, 1H, =CH_2); δ = 4.68 ppm (s, 1H, O*H*); δ = 3.69 ppm (m, 1H, C*H*); δ = 3.04 ppm (m, 2H, CH_2); δ = 1.85 ppm (q, 3H, CH_3); and δ = 1.01 ppm (d, 3H, CH_3).

N-(*tert*-butoxycarbonyl)-*N*'-(6-methacrylamidohexanoyl)hydrazine (MA-AH-NHNH-Boc)

N-(*tert*-butoxycarbonyl)-*N*'-(6-methacrylamidohexanoyl)hydrazine (MA-AH-NHNH-Boc) was prepared in two-step synthesis. First, *N*-methacryloyl-6-amino-hexanoic acid (MA-AH-OH) was prepared by reaction of methacryloyl chloride and 6-aminohexanoic acid in sodium hydroxide aqueous solution,[2, 3] followed by reaction with *tert*-butyl carbazate in THF in the presence of *N*,*N*'-dicyclohexylcarbodiimide (DCC), as reported previously [4]. Melting point: 114 °C. Elemental analysis: calculated C 57.70 %, H 8.33 %, N 13.46 %; Found C 58.66 %, H 8.84 %, N 13.16 %. ^{1}H NMR (DMSO-d_6, 600 MHz): δ = 9.43 ppm (s, 1H, N*H*); δ = 8.63 ppm (s, 1H, N*H*); δ = 7.85 ppm (s, 1H, N*H*); δ = 5.61 ppm (s, 1H, =CH_2); δ = 5.28 ppm (s, 1H, =CH_2); δ = 3.06 ppm (q, 2H, CH_2); δ = 2.04 ppm (t, 2H, CH_2); δ = 1.83 ppm (q, 3H, CH_3); δ = 1.49 ppm (quint, 2H, CH_2); δ = 1.42 ppm (quint, 2H, CH_2); δ = 1.38 ppm (s, 9H, CH_3); and δ = 1.24 ppm (quint, 2H, CH_2).

Chain transfer agent S-2-cyano-2-propyl-S-ethyl trithiocarbonate

The CTA, S-2-cyano-2-propyl-S-ethyl trithiocarbonate (trithio-AIBN), was synthesized as described by Ishitake et al. [5]. Trithio-AIBN was obtained as yellow-orange oil. The HPLC showed a single peak with a retention time of 10.7 min at UV-Vis detection 305 nm. ^{1}H NMR, d: 1.36 (t, 3H, SCH2CH3), d: 1.88 (s, 6H, C(CH3)2CN), d: 3.35 (q, 2H, SCH2CH3). ESIMS: *m/z* (M + Na)+ calculated. for $C_7H_{11}NS_3$ 228.06 found m/z [M+Na]+ 228.16.

Polymer precursor poly(HPMA-*co*-MA-AH-NHNH$_2$) (P1)

The detailed synthesis of polymer precursor poly(HPMA-*co*-MA-AH-NHNH$_2$) (**P1**) was as follows: HPMA (1.5 g, 10.5 mmol), MA-AH-NHNH-Boc (365 mg, 1.2 mmol), and S-2-cyano-2-propyl-S`-ethyl trithiocarbonate (CTA) (13.3 mg, 64.6 μmol) were dissolved in *tert*-butanol (16.3 mL), then mixed with a solution of 2,2'-azo*bis*(4-methoxy-2,4-dimethylvaleronitrile) (V-70) (9.97 mg, 32.3 μmol) in DMA (328 μL) – the mixture contained 0.7 M solution of monomers. The reaction mixture was bubbled with argon and the polymerization was carried out in a thermostat-controlled water bath at 30 °C for 72 h. The polymer was isolated by precipitation into a mixture of dry acetone and dry diethyl ether (2/1, *v/v*; 500 mL) followed by centrifugation at 7800 rpm for 3 min. The crude polymer was filtered off, purified by reprecipitation from methanol, filtered, and dried under vacuum (1.44 g, 77 %). The trithiocarbonate end groups were removed via reaction with an excess of 2,2′-azo*bis*isobutyronitrile (AIBN), as previously described [6]. AIBN (287 mg) was added into a solution of polymer (1.43 g) in dry DMA (11 mL) and bubbled with argon. After 3 h in a thermostat-controlled water bath at 80 °C, the solution was isolated as described above. The precipitate was dried under vacuum, resulting in the polymer with protected hydrazide groups (1.3 g, 91 %). Boc groups were removed in Q-H_2O at 100 °Cas previously described [7]. After 2 h, the solution was freeze-dried, resulting in **P1** with reactive hydrazide groups (1.12 g, 87 %) (**Fig. S1**).

Polymer precursor poly(HPMA-*co*-MA-APMA) (P2)

The polymer precursor **P2** containing amine groups poly(HPMA-*co*-MA-APMA) was prepared analogously by using HPMA and *N*-(3-*tert*-butoxycarbonyl-aminopropyl)methacrylamide (APMA-Boc), as previously described [8] (**Fig. S1**).

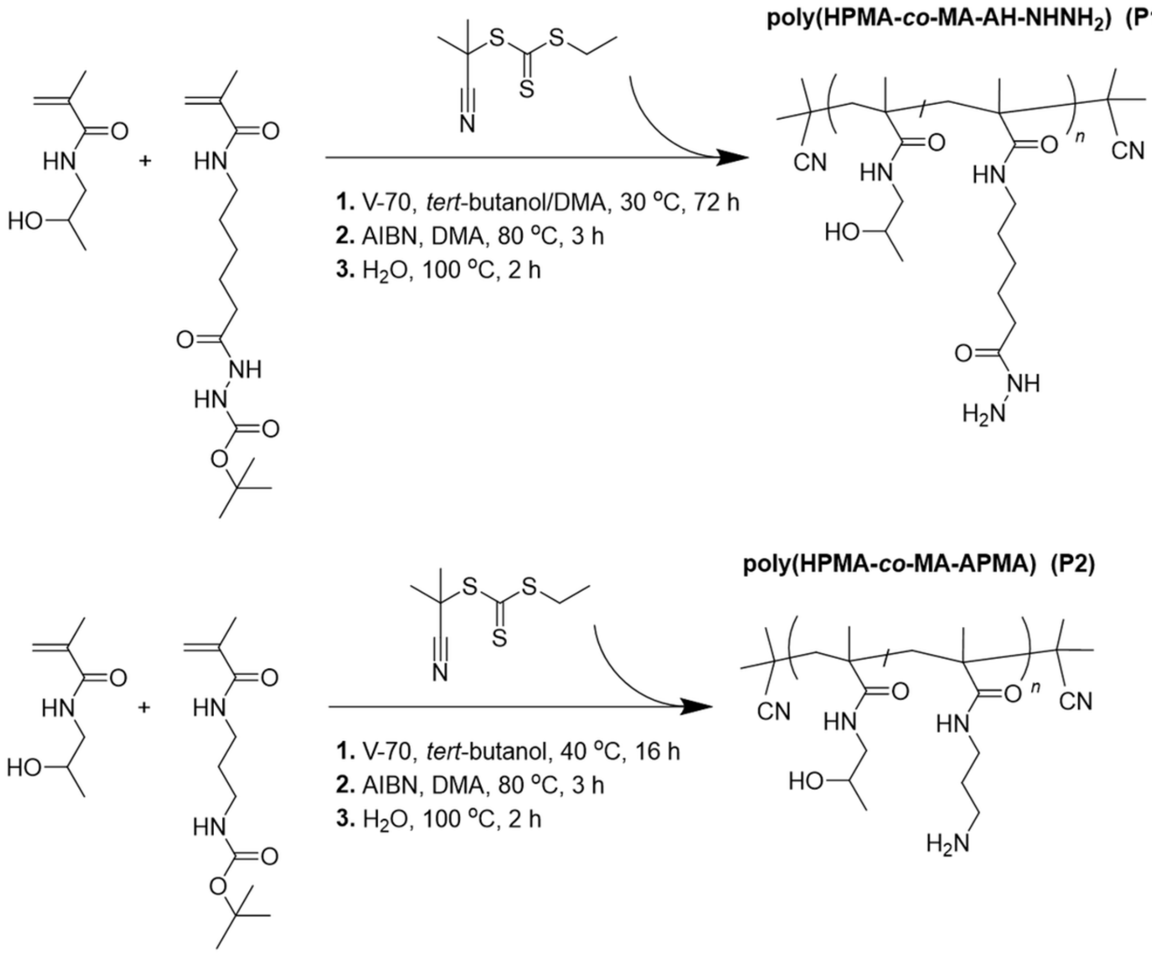

Figure S1. Synthesis of polymer precursors poly(HPMA-*co*-MA-AH-NHNH$_2$) (**P1**) and poly(HPMA-*co*-MA-APMA) (**P2**).

2. Physico-chemical characterization

2.1. Nuclear magnetic resonance of monomers, chain transfer agent, polymer precursors and conjugates

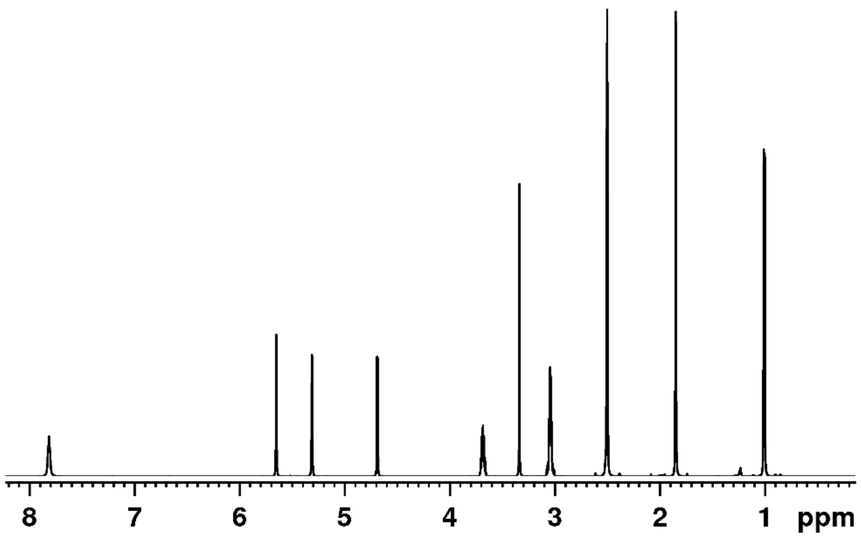

Figure S2.1.1. ^{1}H NMR spectrum of HPMA (600.23 MHz for ^{1}H, DMSO-d_6, 22 °C).

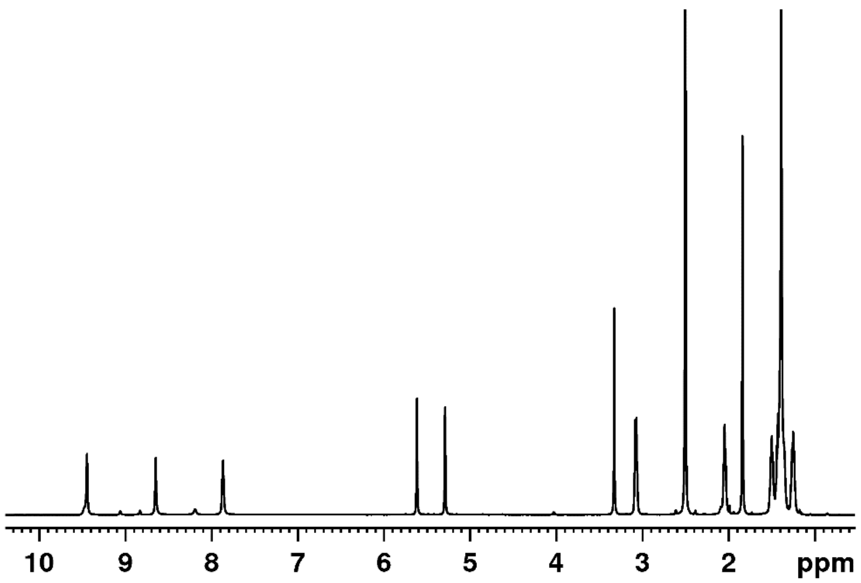

Figure S2.1.2. ^{1}H NMR spectrum of MA-AH-NHNH-Boc (600.23 MHz for ^{1}H, DMSO-d_6, 22 °C).

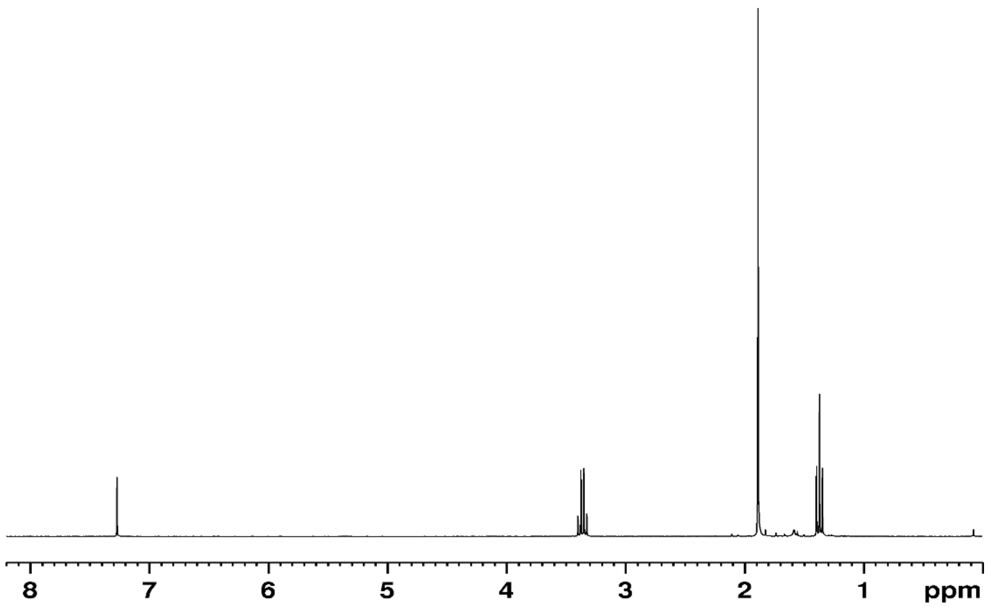

Figure S2.1.3. ^{1}H NMR spectrum of S-2-cyano-2-propyl-S`-ethyl trithiocarbonate (600.23 MHz for ^{1}H, $CDCl_3$, 22 ºC).

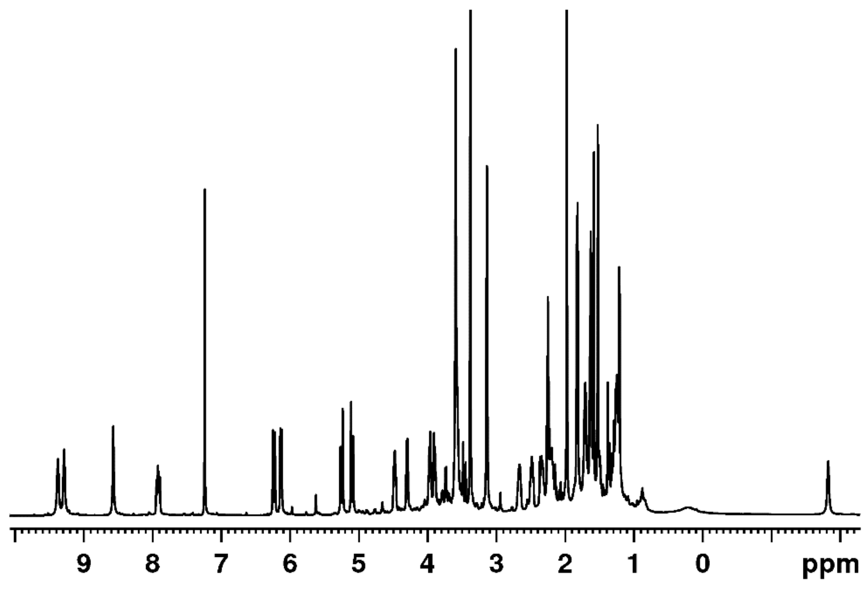

Figure S2.1.4. ^{1}H NMR spectrum of derivative dPyF (600.23 MHz for ^{1}H, $CDCl_3$, 22 ºC).

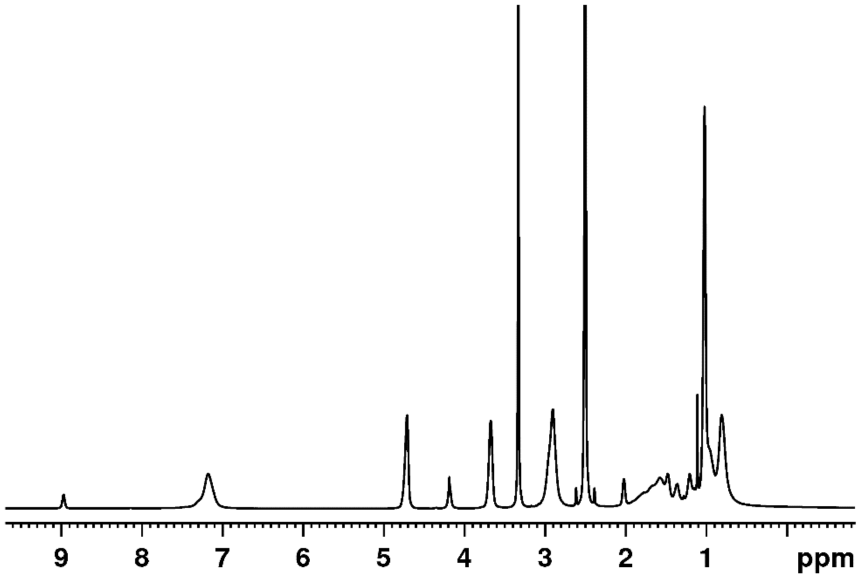

Figure S2.1.5. ^{1}H NMR spectrum of precursor poly(HPMA-*co*-MA-AH-NHNH$_2$) (**P1**) (600.23 MHz for ^{1}H, DMSO-d_6, 22 °C)

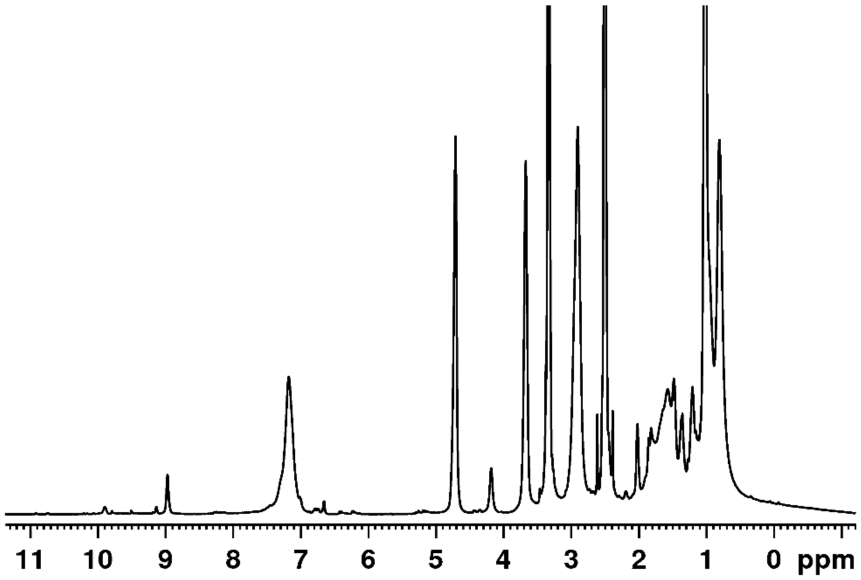

Figure S2.1.6. ^{1}H NMR spectrum of **P-hyd-dPyF** (600.23 MHz for ^{1}H, DMSO-d_6, 22 °C).

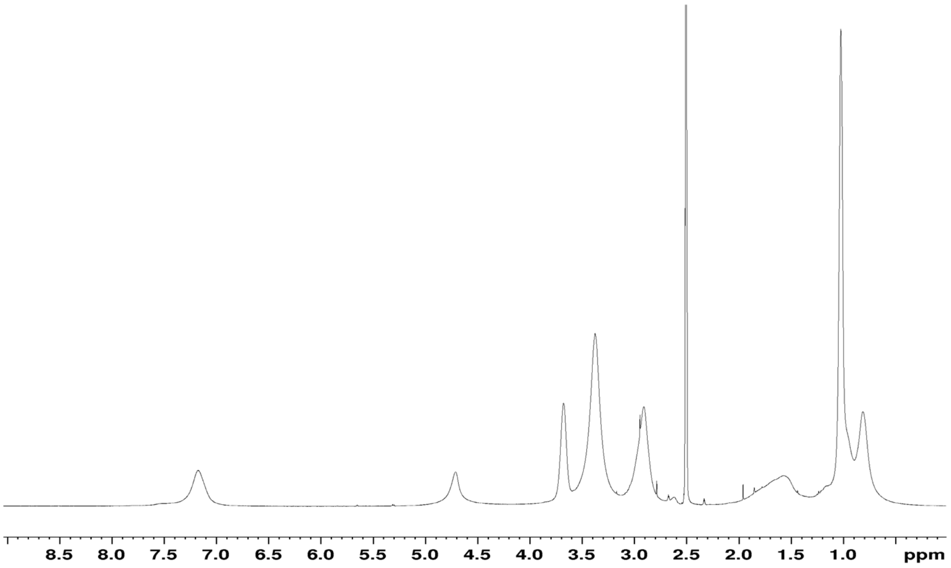

Figure S2.1.7. ^{1}H NMR spectrum of precursor poly(HPMA-*co*-MA-AP-NH_2) (**P2**) (400 MHz for ^{1}H, DMSO-d_6, 22 ºC)

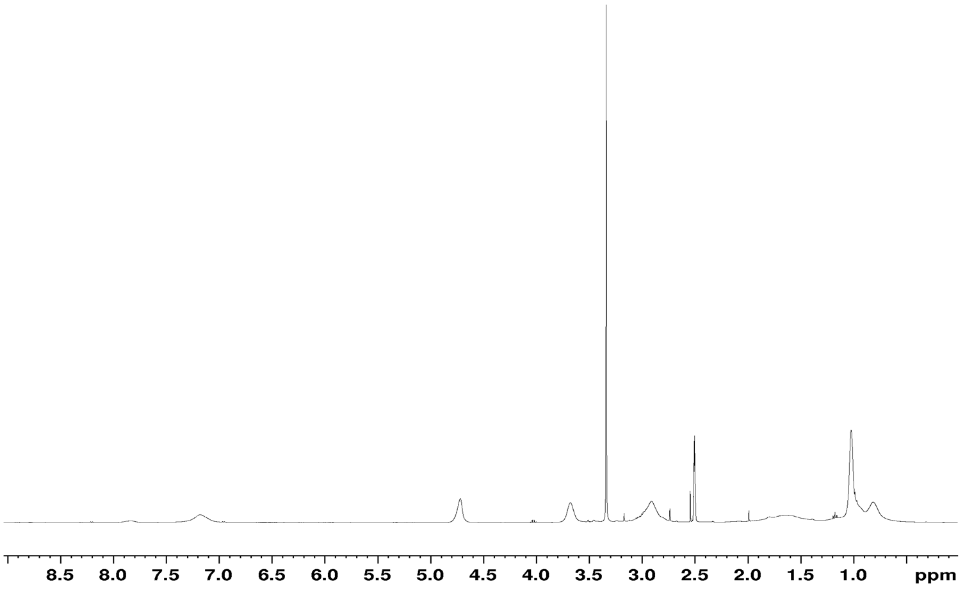

Figure S2.1.8. ^{1}H NMR spectrum of **P-amide-PyF** (400 MHz for ^{1}H, DMSO-d_6, 22 ºC).

2.2. Field flow fractionation and Size exclusion chromatography

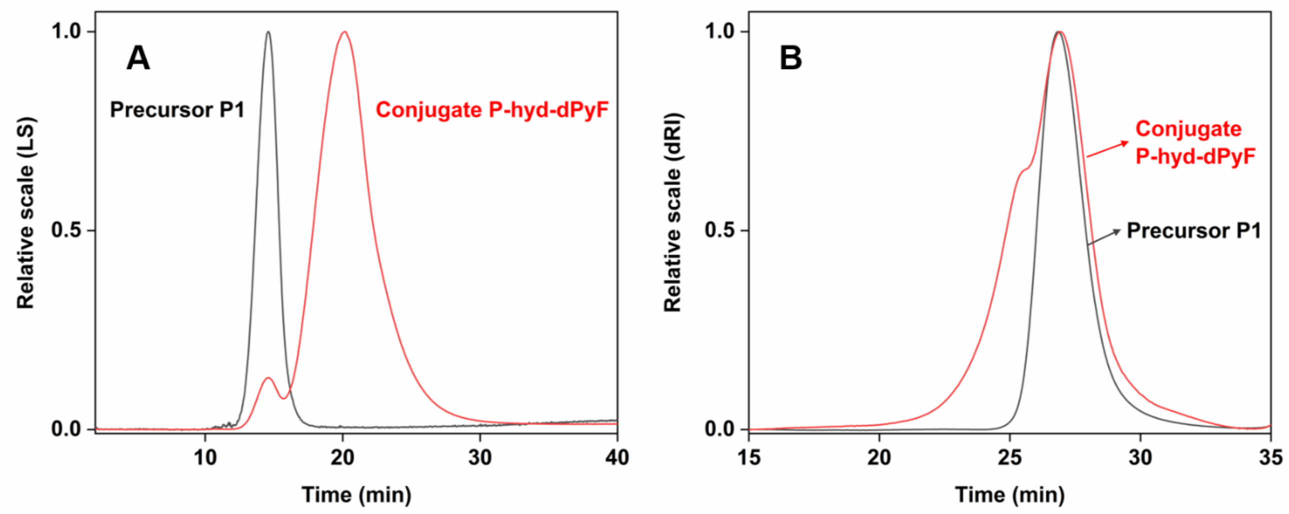

Figure S2.2. Polymer precursor **P1** and polymer conjugate **P-hyd-dPyF**: **(A)** FFF chromatograms in PBS (pH 7.4) at 1.1 mg·mL^{-1}; **(B)** SEC chromatograms in DMF + LiBr (0.5 g L^{-1}).

2.3. UV–Vis spectrophotometry

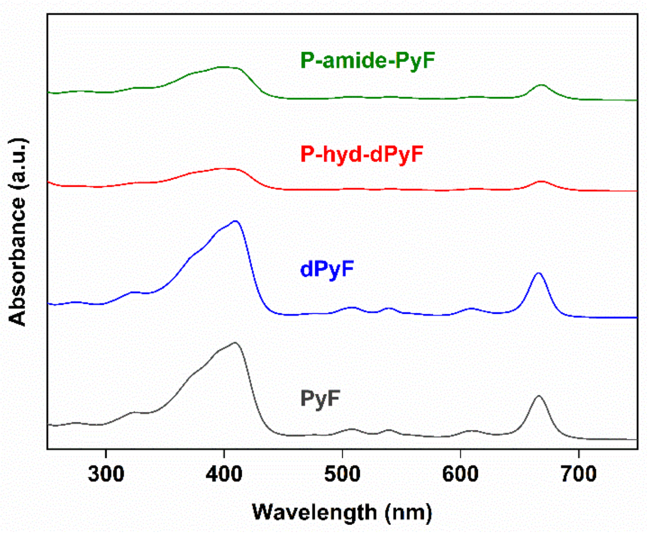

Figure S2.3. UV-Vis spectra of pure PyF, derivative dPyF, conjugate **P-hyd-dPyF**, and conjugate **P-amide-PyF** at 0.1 mg mL^{-1} PyF or dPyF equivalent in methanol.

2.4. TEM microscopy

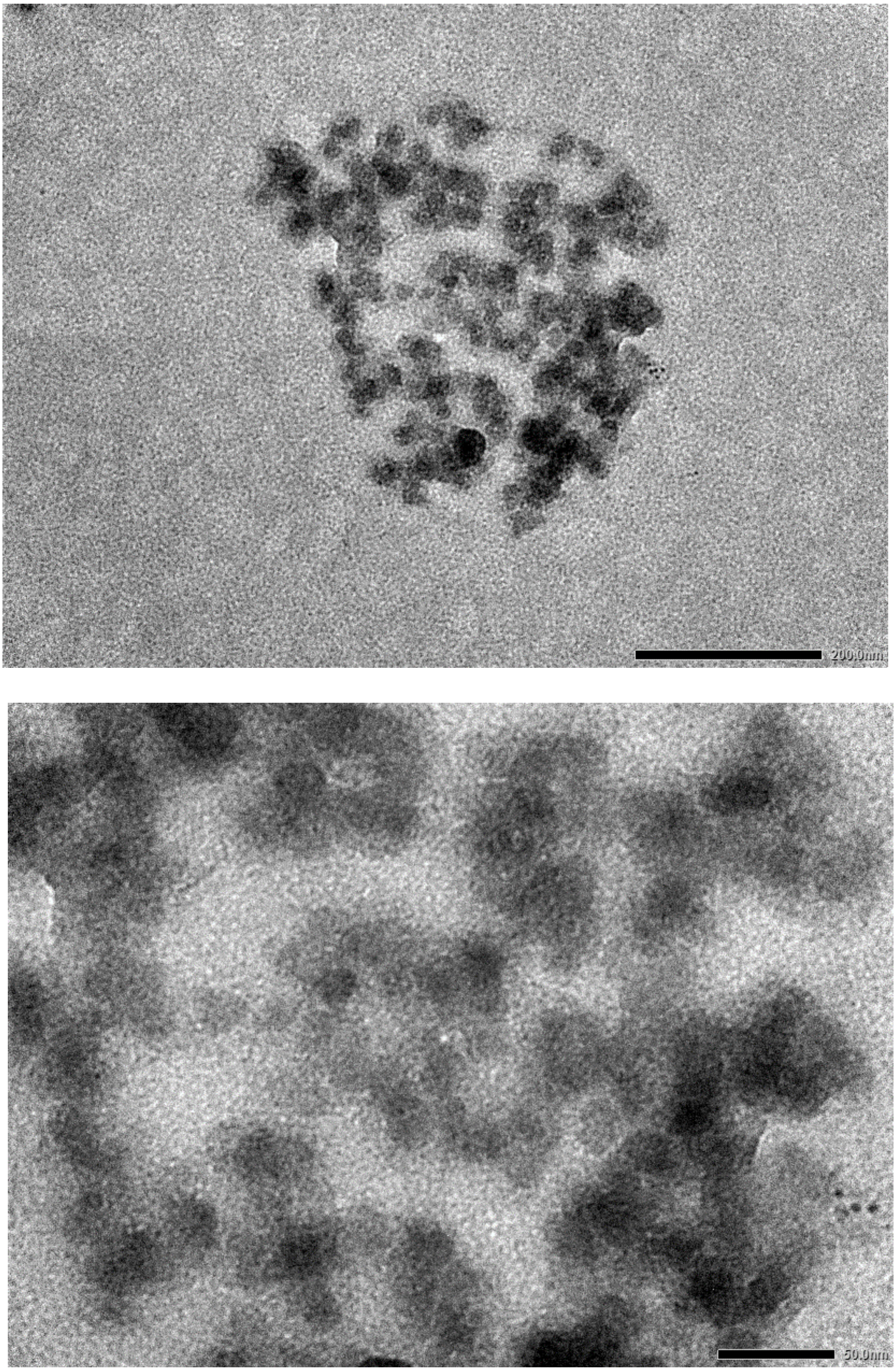

Figure S2.4. TEM microscopy of conjugate **P-hyd-dPyF**

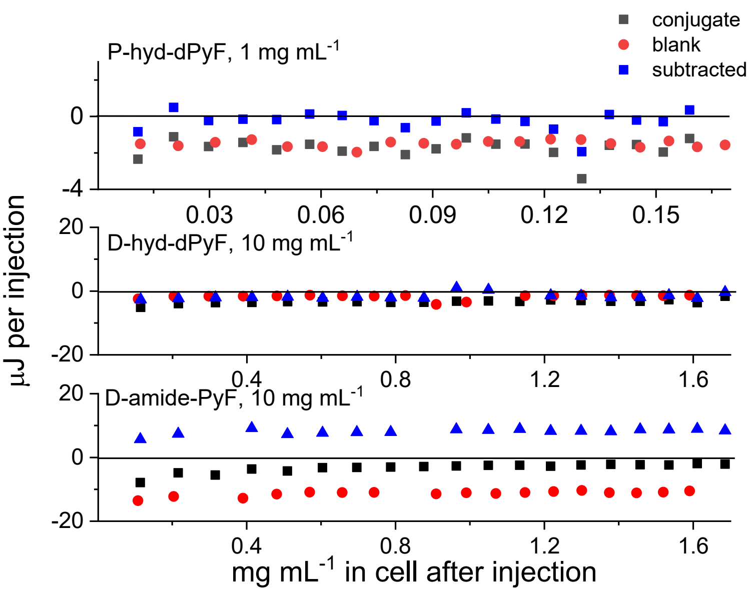

Figure S2.5. Heat of dilution of **P1**, **P2**, **P-amide-PyF** and **P-hyd-dPyF** polymers measured by ITC in PBS at 37°C

3. *In vitro* PDT effect, *in vivo* tissue distribution and PDT antitumor activity

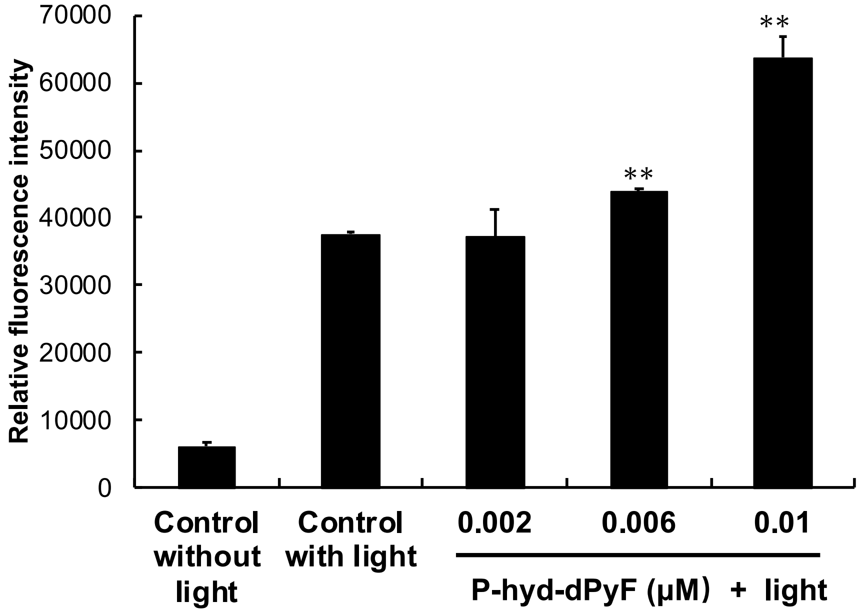

Figure S3.1. Intracellular ROS generation after PDT using **P-hyd-dPyF** treatment. See the manuscript for details. The data represent mean ± SD, n = 4.

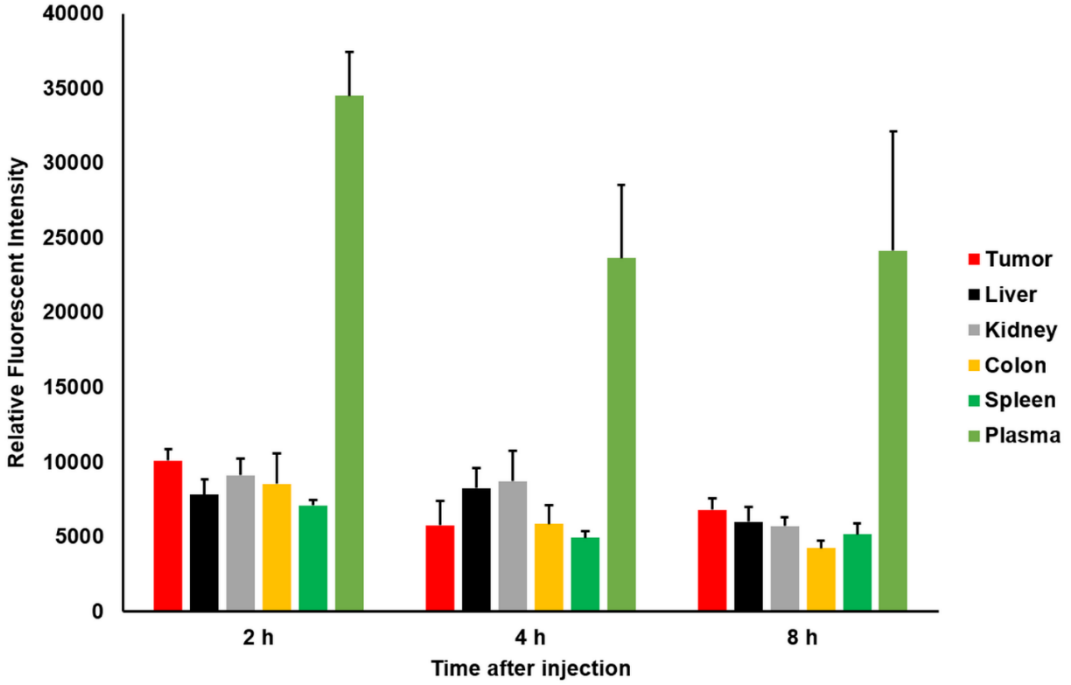

Figure S3.2. Tissue distribution of **P-hyd-dPyF** (5 mg kg^{-1}, dPyF equivalent) at different times after intravenous injection. The mouse sarcoma S180 solid tumor model was used. See the manuscript for details. The data represent mean ± SD, n = 6-8.

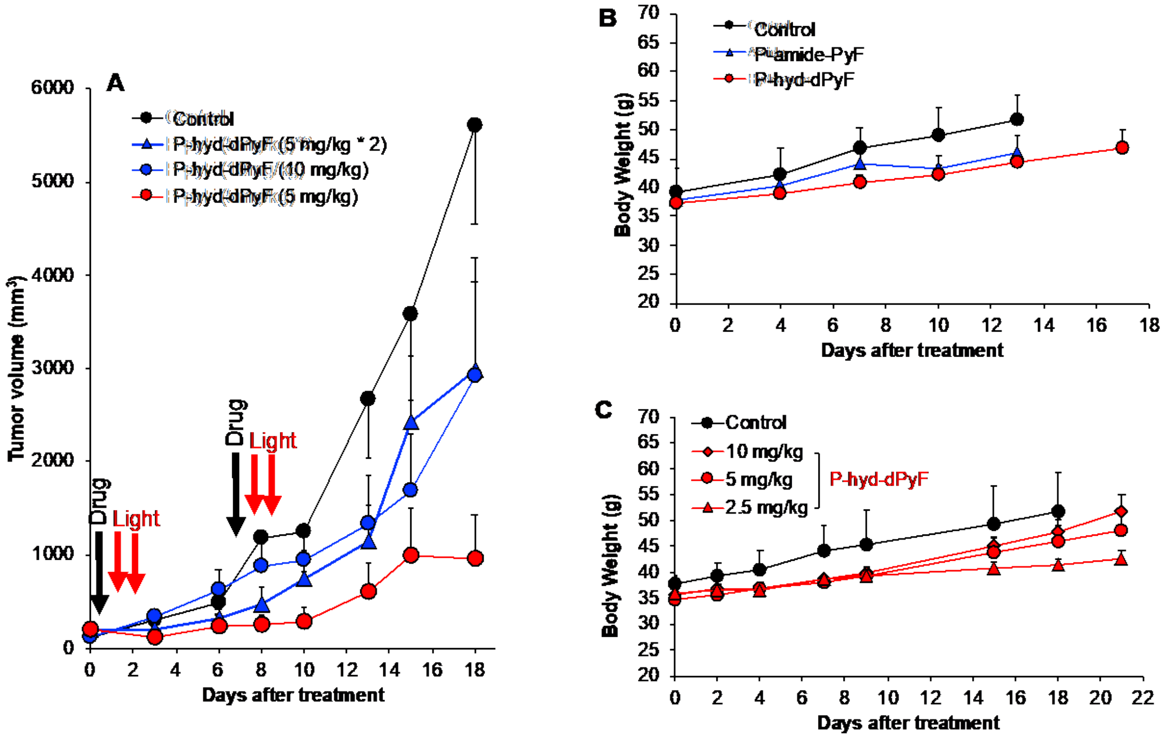

Figure S3.3. *In vivo* PDT effect of **P-hyd-dPyF (A)**, and body weight changes of the mice after the treatment **(B, C)**. The mouse sarcoma S180 solid tumor model was used. See the manuscript for details. The data represent mean ± SD, n = 6-8.

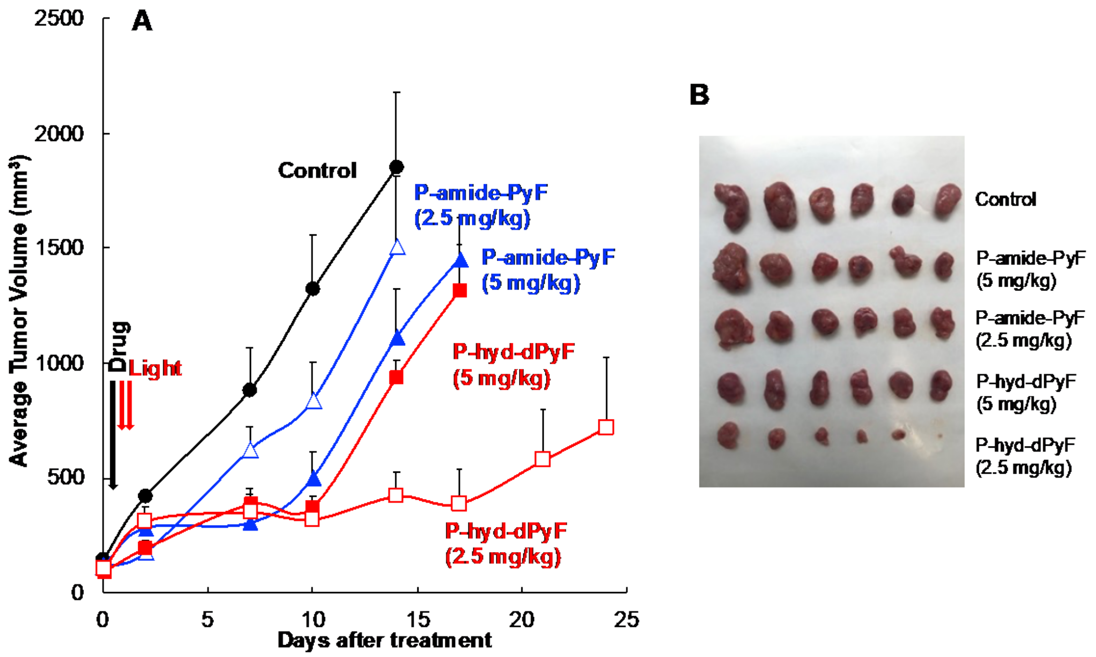

Figure S3.4. *In vivo* PDT effect of **P-hyd-dPyF** in colon cancer C26 bearing mice. The changes of tumor volumes were shown in (**A**), and the image of tumors of each group was shown in (**B**). See the manuscript for details. The data represent mean ± SD, n = 6-8.

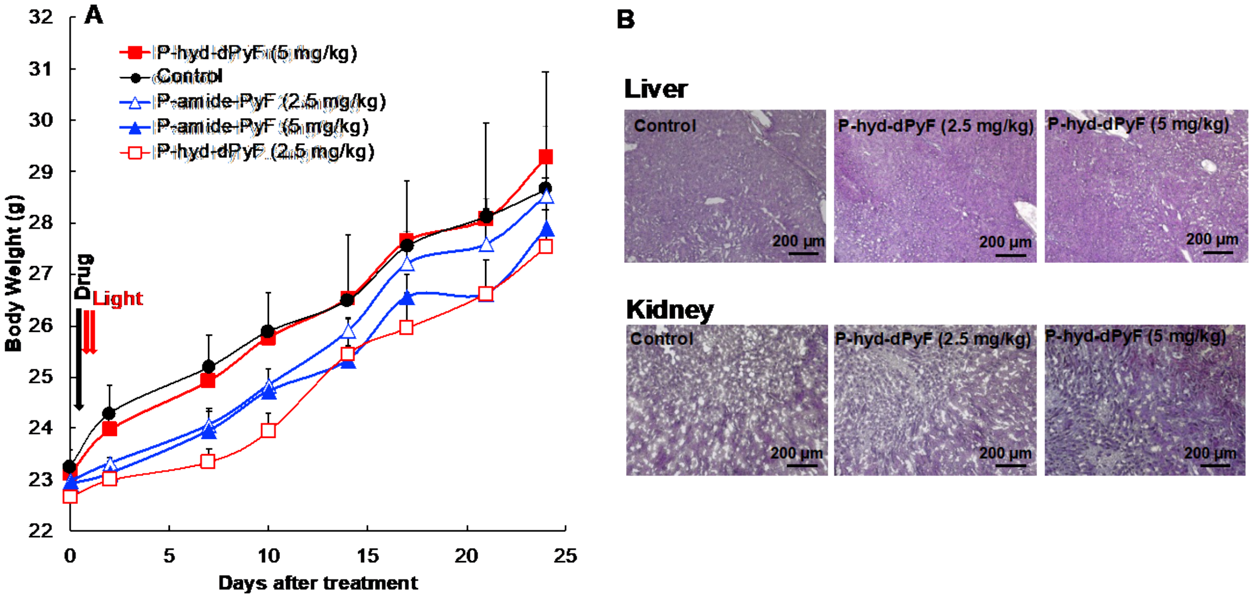

Figure S3.5. Evaluation of side effects of PDT using **P-hyd-dPyF** in colon cancer C26 bearing mice. The changes of body weight were shown in (**A**), and the histological examination (H&E staining) of major organs, i.e., the liver and kidney were shown in (**B**). See the manuscript for details. The data represent mean ± SD, n = 6-8.

4. References

1. Chytil P, Etrych T, Kříž J, Šubr V, Ulbrich K. *N*-(2-hydroxypropyl)methacrylamide-based polymer conjugates with ph-controlled activation of doxorubicin for cell-specific or passive tumour-targeting. Synthesis by raft polymerisation and physicochemical characterisation. Eur J Pharm Sci. 2010; 41: 473-82.
2. Drobník J, Kopeček J, Labský J, Rejmanová P, Exner J, Saudek V, et al. Enzymatic cleavage of side chains of synthetic water-soluble polymers. Makromol Chem. 1976; 177: 2833-48.
3. Etrych T, Chytil P, Jelínková M, Říhová B, Ulbrich K. Synthesis of hpma copolymers containing doxorubicin bound via a hydrazone linkage. Effect of spacer on drug release and in vitro cytotoxicity. Macromol Biosci. 2002; 2: 43-52.
4. Ulbrich K, Etrych T, Chytil P, Jelínková M, Říhová B. Antibody-targeted polymer-doxorubicin conjugates with ph-controlled activation. J Drug Target. 2004; 12: 477-89.
5. Ishitake K, Satoh K, Kamigaito M, Okamoto Y. Stereogradient polymers formed by controlled/living radical polymerization of bulky methacrylate monomers. Angew Chem Int Ed. 2009; 48: 1991-4.
6. Perrier S, Takolpuckdee P, Mars CA. Reversible addition-fragmentation chain transfer polymerization: End group modification for functionalized polymers and chain transfer agent recovery. Macromolecules. 2005; 38: 2033-6.
7. Koziolová E, Kostka L, Kotrchová L, Šubr V, Konefal R, Nottelet B, et al. Hpma-based linear, diblock and star-like polymer drug carriers: Advanced process for their simple production. Biomacromolecules. 2018; 19: 4003-13.
8. Fang J, Šubr V, Islam W, Hackbarth S, Islam R, Etrych T, et al. *N*-(2-hydroxypropyl)methacrylamide polymer conjugated pyropheophorbide-a, a promising tumor-targeted theranostic probe for photodynamic therapy and imaging. Eur J Pharm Biopharm. 2018; 130: 165-76.
